# Genetic Mapping, Candidate Gene Identification and Marker Validation for Host Plant Resistance to the Race 4 of *Fusarium oxysporum* f. sp. *cubense* Using *Musa acuminata* ssp. *malaccensis*

**DOI:** 10.3390/pathogens12060820

**Published:** 2023-06-09

**Authors:** Andrew Chen, Jiaman Sun, Altus Viljoen, Diane Mostert, Yucong Xie, Leroy Mangila, Sheryl Bothma, Rebecca Lyons, Eva Hřibová, Pavla Christelová, Brigitte Uwimana, Delphine Amah, Stephen Pearce, Ning Chen, Jacqueline Batley, David Edwards, Jaroslav Doležel, Peter Crisp, Allan F. Brown, Guillaume Martin, Nabila Yahiaoui, Angelique D’Hont, Lachlan Coin, Rony Swennen, Elizabeth A. B. Aitken

**Affiliations:** 1 School of Agriculture and Food Science, The University of Queensland, Brisbane, QLD 4067, Australia; jiamansun@hotmail.com (J.S.); l.mangila@uq.net.au (L.M.); r.lyons@uq.edu.au (R.L.); n.chen@uq.edu.au (N.C.); p.crisp@uq.edu.au (P.C.); e.aitken@uq.edu.au (E.A.B.A.); 2 School of Life Science, Jiaying University, Meizhou 514015, China; 3 Department of Plant Pathology, Stellenbosch University, Stellenbosch 7600, South Africa; altus@sun.ac.za (A.V.); diane@sun.ac.za (D.M.); sherylb@sun.ac.za (S.B.); 4 Department of Biology, Duke University, Durham, NC 27708-0338, USA; yucong.xie@duke.edu; 5 Institute of Experimental Botany of the Czech Academy of Sciences, Centre of the Region Haná for Bio-Technological and Agricultural Research, CZ-77900 Olomouc, Czech Republic; hribova@ueb.cas.cz (E.H.); christelova@ueb.cas.cz (P.C.); dolezel@ueb.cas.cz (J.D.); 6 International Institute of Tropical Agriculture, Kampala P.O. Box 7878, Uganda; b.uwimana@cgiar.org (B.U.); r.swennen@cgiar.org (R.S.); 7 International Institute of Tropical Agriculture, Ibadan PMB 5320, Nigeria; d.amah@cgiar.org; 8 Sustainable Soils and Crops, Rothamsted Research, Harpenden, Hertfordshire AL5 2JQ, UK; stephen.pearce@rothamsted.ac.uk; 9 School of Biological Sciences, The University of Western Australia, Perth, WA 6009, Australia; jacqueline.batley@uwa.edu.au (J.B.); dave.edwards@uwa.edu.au (D.E.); 10 The Centre for Applied Bioinformatics, University of Western Australia, Crawley, Perth, WA 6009, Australia; 11 International Institute of Tropical Agriculture, Arusha P.O. Box 447, Tanzania; a.brown@cgiar.org; 12 CIRAD, UMR AGAP Institut, F-34398 Montpellier, France; guillaume.martin@cirad.fr (G.M.); nabila.yahiaoui@cirad.fr (N.Y.); angelique.dhont@cirad.fr (A.D.); 13 UMR AGAP Institut, Université de Montpellier, CIRAD, INRAE, Institut Agro, F-34398 Montpellier, France; 14 Department of Microbiology and Immunology, Peter Doherty Institute for Infection and Immunity, University of Melbourne, Melbourne, VIC 3004, Australia; lachlan.coin@unimelb.edu.au; 15 Division of Crop Biotechnics, Laboratory of Tropical Crop Improvement, Katholieke Universiteit Leuven, 3001 Leuven, Belgium

**Keywords:** banana, fine mapping, quantitative trait locus, *Musa acuminata* ssp. *malaccensis*, Fusarium wilt, *Fusarium oxysporum* f. sp. *cubense*, tropical race 4, subtropical race 4, marker-assisted selection, resistance gene expression, receptor-like kinase, RNAseq

## Abstract

Fusarium wilt of banana is a devastating disease that has decimated banana production worldwide. Host resistance to *Fusarium oxysporum* f. sp. *Cubense* (*Foc*), the causal agent of this disease, is genetically dissected in this study using two *Musa acuminata* ssp. *Malaccensis* segregating populations, segregating for *Foc* Tropical (TR4) and Subtropical (STR4) race 4 resistance. Marker loci and trait association using 11 SNP-based PCR markers allowed the candidate region to be delimited to a 12.9 cM genetic interval corresponding to a 959 kb region on chromosome 3 of ‘DH-Pahang’ reference assembly v4. Within this region, there was a cluster of pattern recognition receptors, namely leucine-rich repeat ectodomain containing receptor-like protein kinases, cysteine-rich cell-wall-associated protein kinases, and leaf rust 10 disease-resistance locus receptor-like proteins, positioned in an interspersed arrangement. Their transcript levels were rapidly upregulated in the resistant progenies but not in the susceptible F_2_ progenies at the onset of infection. This suggests that one or several of these genes may control resistance at this locus. To confirm the segregation of single-gene resistance, we generated an inter-cross between the resistant parent ‘Ma850’ and a susceptible line ‘Ma848’, to show that the STR4 resistance co-segregated with marker ‘28820’ at this locus. Finally, an informative SNP marker 29730 allowed the locus-specific resistance to be assessed in a collection of diploid and polyploid banana plants. Of the 60 lines screened, 22 lines were predicted to carry resistance at this locus, including lines known to be TR4-resistant, such as ‘Pahang’, ‘SH-3362’, ‘SH-3217’, ‘Ma-ITC0250’, and ‘DH-Pahang/CIRAD 930’. Additional screening in the International Institute for Tropical Agriculture’s collection suggests that the dominant allele is common among the elite ‘Matooke’ NARITA hybrids, as well as in other triploid or tetraploid hybrids derived from East African highland bananas. Fine mapping and candidate gene identification will allow characterization of molecular mechanisms underlying the TR4 resistance. The markers developed in this study can now aid the marker-assisted selection of TR4 resistance in breeding programs around the world.

## 1. Introduction

Bananas (*Musa* spp.) are an important horticulture crop, typically consumed as a fruit or staple food, and they are cultivated in the tropical and subtropical regions around the world. *Musa* spp. were domesticated in Southeast Asia and Melanesia, and hybridisation involving mainly A (*Musa acuminata*) and B (*Musa balbisiana*) genome progenitors gave rise to most of the domesticated forms of the dessert and plantain bananas we see today [1,2,3]. *Musa acuminata* have been divided into multiple subspecies [4,5], and hybridisation among them resulted in edible diploids. Restitution of the gametes at meiosis led to the formation of triploid cultivars [1,6,7].

Fusarium wilt of banana (FWB), also known as Panama disease, is one of the most devastating diseases affecting banana plants. The global epidemics owing to FWB have put major constraints on banana production both historically and at the present time [8,9]. The causal agent for this disease is the soil-borne fungus *Fusarium oxysporum* f. sp. *cubense* (*Foc*). *Foc* can be classified into a race structure, reflecting its banana host range [10,11,12,13] and unique vegetative compatibility groups (VCGs). *Foc* race 1 was the cause of the pandemic that decimated the triploid cultivar ‘Gros Michel’ (genome AAA) during the last century. Its replacement, the ‘Cavendish’ banana, is resistant to *Foc* race 1. Cavendish bananas are now the dominant cultivar on the market, accounting for more than 40% of the 124 M tonnes of world banana production in 2021 [14], with export markets accounting for approximately 15% of the total production [15].

During the 1990s, a previously unknown race, the tropical race 4 (TR4) of FWB, emerged and decimated Cavendish plantations around the world [16,17]. According to the range of the banana subgroups affected, TR4 strains are collectively classified by subtropical race 4 (STR4) as members of race 4. Vegetative compatibility grouping (VCG) and multi-loci molecular phylogeny have provided distinction between the two groups of isolates [11,13,18,19]. STR4 can infect Cavendish plants under subtropical conditions, whereas TR4 is virulent on all Cavendish and many other banana cultivars under both tropical and subtropical conditions [20]. So far, TR4 has significantly curtailed banana production in Australia [21], China [22], Indonesia [23], Malaysia [24], the Philippines [19,25], Jordan [26], Israel and other Middle East regions [27], India [28], Mayotte [29], and Africa [30] and has spread to locations as far as Colombia and Peru [31,32]. The disease poses a major threat to banana production, limiting the selection of cultivars and the land suitable for commercial production and, at the same time, putting constraints on food security of smallholders.

*Foc* infects banana plants through the roots then travels through the vascular vessels to colonise the rhizome and the pseudostem of susceptible plants [33,34]. Symptoms are manifested as localised necrotic lesions in and around the vascular vessels. Eventually the mycelia travel up through the xylem and establish themselves in the aerial parts of the plants. Extensive fungal colonisation blocks the water-conducting vessels of the xylem, restricting water and nutrient supplies to the plant. This leads to wilting of the leaves and eventually kills the plant. Once *Foc* is disseminated in infected soil, it can remain in the soil for decades, surviving as chlamydospores on infected planting material or as endophytes on alternative weed hosts and spreading through the movement of contaminated water and soil [35,36]. Disease control strategies have focused on deterrence through biosecurity measures [16], providing clean planting materials [37] and biocontrol agents, such as *Tricoderma* spp. or endophytic *F. oxysporum* spp. [20,38,39].

Host genetic resistance to *Foc* provides a long-term solution for the management of the disease. *Foc* race 4 type resistance has been detected in both wild and cultivated banana plants [33,40,41,42,43,44,45]. Wild relatives or cultivated diploid varieties, including *M. acuminata* ssp. *malaccensis* ‘Pahang’, ‘DH-Pahang’, *M. acuminata* ssp. *burmannica* ‘Calcutta 4’, *M. itinerans*, cv. ‘Tuu Gia’, and cv. ‘Rose’, are highly resistant to *Foc* TR4 [41,45,46]. Inter- and intra-specific hybrids, such as ‘FHIA21’, ‘FHIA25’, ‘SH3142’, as well as all tested plantains and East African highland bananas (EAHBs), also exhibit high levels of TR4 resistance [43,45]. The Cavendish somaclones ‘GCTCV’ carry varying levels of TR4 resistance [33,43,45]. In some cases, TR4 resistance or susceptibility expressed by some of these somaclones appeared to be dependent on the inoculum dosage as well as the environment [42,43,45].

Forward genetic approaches have led to the identification of genes controlling plant yield and development, as well as biotic and abiotic stress tolerance [47]. Genetic mapping typically identifies major genes that control a large percentage of the trait variations [48]. Such genes are useful for developing molecular markers to select favourable alleles in breeding programs [49].

In banana, forward genetics have not been performed frequently due to experimental constraints associated with sterility, polyploidy, long life cycles in population development, and phenotypic assessments [50]. Linkage maps have been traditionally constructed using restriction fragment length polymorphism (RFLP), isozymes, random amplified polymorphic DNA (RAPD) [51], microsatellites or simple sequence repeats (SSRs), and amplified fragment length polymorphisms (AFLPs) [52] on *M. acuminata* ssp. *banksii*- and *M. acuminata* ssp. *malaccensis*-derived populations. However, these markers are not easily transferable to other populations, and large segregation distortion has been observed [51]. More recently, diversity array technology (DArT) has been deployed for high throughput genotyping in *Musa* [53]. DArTseq, a powerful genotyping-by-sequencing (GBS) approach to generate high-density linkage maps, has been successfully used for genotyping large segregating populations of diploid and triploid *Musa* spp. [54,55,56].

Previously, we used flow cytometry and simple sequence repeat genotyping to show that wild lines of *Musa* spp. contained a diploid genome and were taxonomically characterised as *Musa acuminata* ssp. *malaccensis* [57]. These *M. acuminata* ssp. *malaccensis* lines were resistant to both STR4 and TR4 [57,58], and they were heterozygous for single-gene resistance, with resistance dominant over susceptibility. A quantitative trait-locus-by-sequencing (QTL-seq) approach was used to identify a major locus on chromosome 3 conferring resistance to STR4 [57]. This QTL is distinct to the QTL identified on chromosome 10 for race 1 and TR4 resistance [54]. Genome ancestry analysis on our lines showed that the region on chromosome 3 had a *M. acuminata* ssp. *malaccensis* origin [57], making this region ideal for gene isolation using the *M. acuminata* ssp. *malaccensis* reference genome [46].

In this study, we performed genetic mapping in the chromosome 3 QTL region by screening a self-derived F_2_ population with SNP-based cleaved amplified polymorphism sequence (CAPS) markers. Individuals carrying recombination events were tested against both STR4 and TR4 strains to define and limit the candidate region. One marker carried an informative SNP that allowed chromosome 3-specific resistance to be assayed in 132 *Musa* accessions, including the core *M. acuminata* ssp. *malaccensis* collection from the International Musa Germplasm Transit Centre (ITC), as well as a comprehensive collection of diploid and polyploid genotypes from the International Institute for Tropical Agriculture (IITA) in Nigeria and Uganda. The validation of this marker will allow marker-assisted selection of TR4 and STR4 resistance to be deployed in breeding programs around the world.

## 2. Results

### 2.1. Foc-STR4 Phenotypes and Population Development

**Figure 1:**
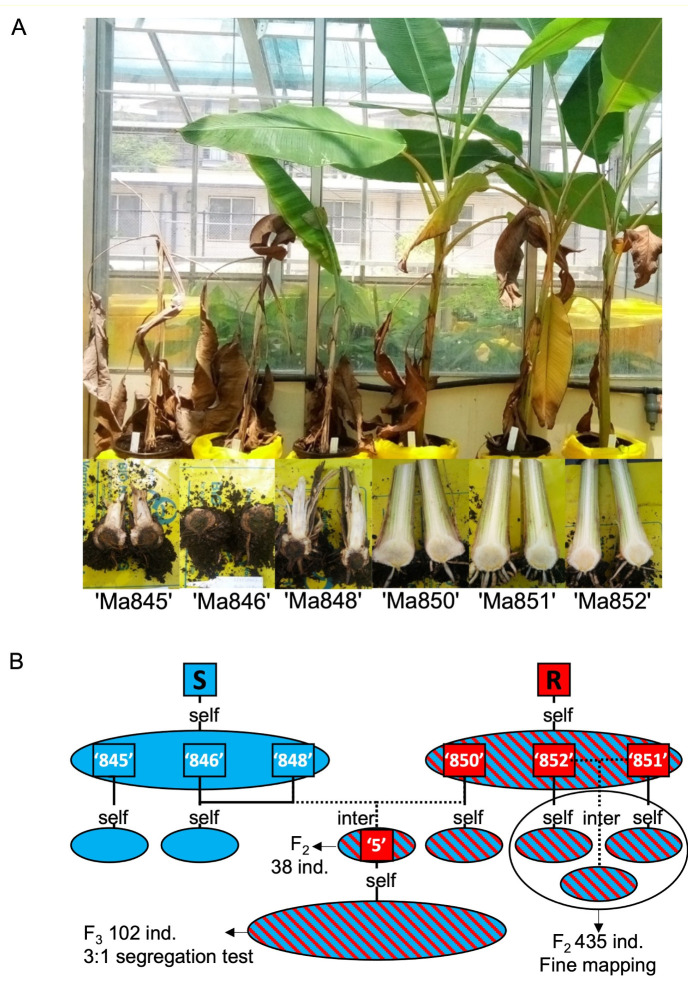
*Foc*-STR4 resistance or susceptibility in the diploid *M. acuminata* ssp. *malaccensis* parents ‘Ma845’, ‘Ma846’, ‘Ma848’, ‘Ma850’, ‘Ma581’, and ‘Ma852’ and the F_2_ population development. (**A**) Representative plants of six genotypes following infection with *Foc*-STR4. *Foc*-STR4-susceptible individuals ‘Ma845’, ‘Ma846’, and ‘Ma848’ displayed vascular wilting and plant death, and brown discolourations were associated with the colonisation of the fungus inside the rhizomes. The ‘Ma850’, ‘Ma851’, and ‘Ma852’ parents were completely resistant to *Foc*-STR4 and did not show any internal or external symptoms. (**B**) The development of *Musa acuminata* ssp. *malaccensis* populations used in this study. The ‘R’ progenitor is the original *Foc* race 4-resistant parent which gave rise after selfing to three F_1_ plants, ‘Ma850’, ‘Ma851’, and ‘Ma852’, segregating for both *Foc*-TR4 and *Foc*-STR4 resistance. A susceptible ‘S’ progenitor, that was not related to the ‘R’ progenitor, gave rise to three self-crossed progenies, ‘Ma845’, ‘Ma846’, and ‘Ma848’, all of which were *Foc* race 4-susceptible. The genetic analysis carried out in this study used self-derived F_2_ progenies of Ma851 and Ma852 as well as progenies derived from an inter-cross between the two (Population 1). The segregation of resistance was further validated using an inter-cross between ‘Ma850’ and ‘Ma848’ (Population 2). The F_2_ line #5 from this cross was selfed to generate an F_3_ population segregating for STR4 resistance. Rectangles indicate parental lines. Ovals indicate progenies derived from the same parent(s). Parents are coloured according to resistant (red) or susceptible (blue) *Foc* race 4 phenotypes. Progenies (ovals) are shaded blue to indicate the absence of resistance amongst all progenies tested or exhibit red/blue stripes to indicate the segregation of *Foc* race 4 resistance within the population. Solid lines indicate self-cross pollinations. A dashed line indicates an inter-cross.

Three STR4-resistant (‘Ma850’, ‘Ma851’, and ‘Ma852’) and three STR4-susceptible (‘Ma845’, ‘Ma846’, and ‘Ma848’) *M. acuminata* ssp. *malaccensis* F_1_ parental lines were derived from two independent progenitors (Figure 1). From each parent, 20–30 self-derived progenies were previously tested against both STR4 and TR4. The progenies of ‘Ma850’, ‘Ma851’, and ‘Ma852’ were segregated for single-gene resistance to both STR4 and TR4 at a 3R:1S ratio, whereas the progenies of ‘Ma845’, ‘Ma846’, and ‘Ma848’ were uniformly susceptible to both races [58]. Subsequently, four F_2_ populations segregating for *Foc*-STR4 resistance were developed (Figure 1B). ‘Population 1’ comprised two self- and one inter-cross between the R parents ‘Ma851’ and ‘Ma852’, which are known to segregate for STR4 and TR4 resistance. ‘Population 2’ was derived from an inter-cross between ‘Ma850’ and ‘Ma848’ (Figure 1B). A total of 435 F_2_ and 102 F_3_ individuals from Population 1 and Population 2, respectively, were obtained from embryo germination in tissue culture and then multiplied to sufficient numbers for phenotyping.

### 2.2. Genetic Mapping

**Figure 2:**
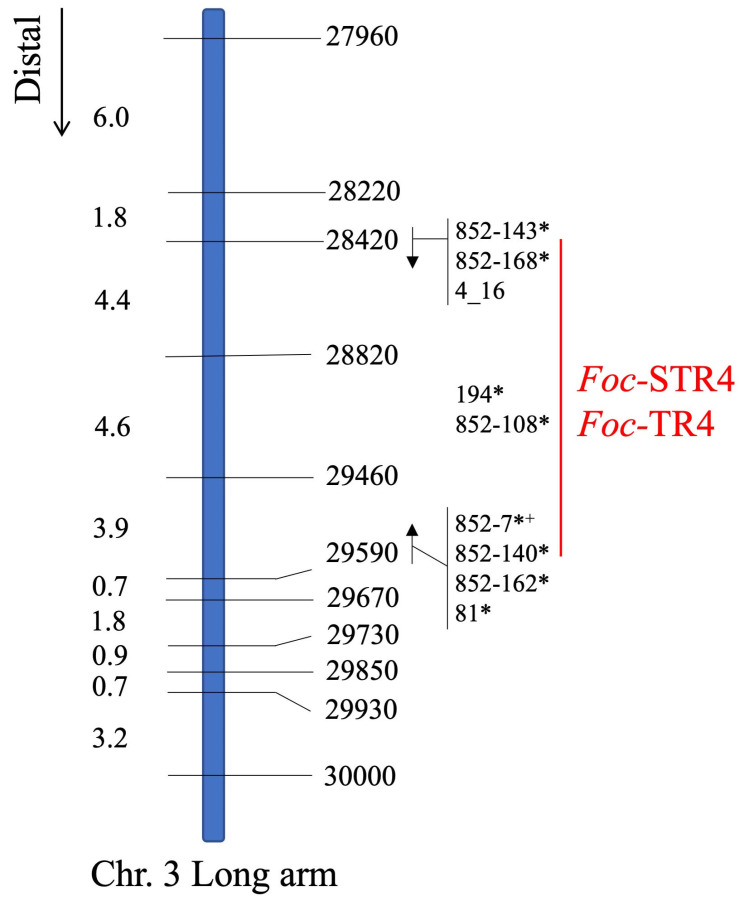
A genetic map constructed using CAPS markers developed in the QTL region at the distal end of the long arm of chromosome 3. The marker names correspond to the numeric part of the ‘DH-Pahang’ v1 gene names. The centiMorgan (cM) distance between markers on the left is calculated from 435 F_2_ individuals derived from the self-crosses of ‘Ma851’ × ‘Ma851’ and ‘Ma852’ × ‘Ma852’ and the inter-cross of ‘Ma851’ × ‘Ma852’, collectively referred to as Population 1. The candidate region is mapped to a 12.9 cM genetic interval between markers 28420 and 29590. The *Foc*-STR4/*Foc*-TR4 resistance locus is highlighted in red. This locus is defined by multiple critical lines carrying recombination events between markers 28420 and 28820 and between markers 29460 and 29590. The markers most closely linked to the locus are 28820 and 29460. The directions of the marker–trait association are indicated with an arrow. All lines were tested against *Foc*-STR4. Asterisks (*) indicates that these lines were additionally tested against *Foc*-TR4. Plus (+) indicates that the *Foc*-TR4 phenotype of this line was not in agreement with all the other lines tested at the same recombined position.

**Figure 3:**
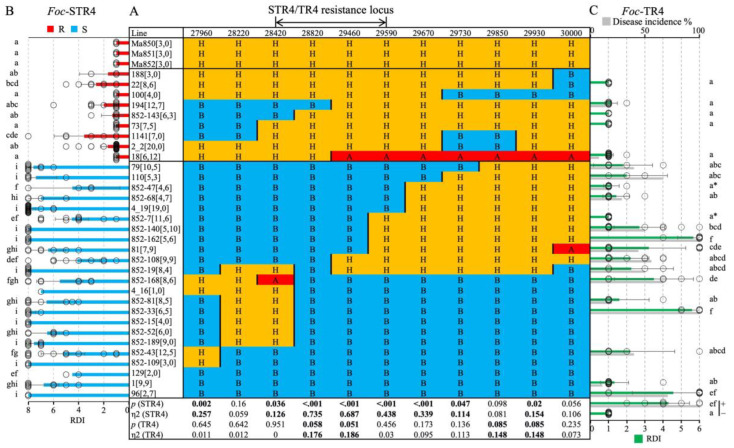
Fine mapping of the STR4/TR4 resistance locus. (**A**) A genetic map constructed using mostly homozygous B/H (B: marker allele homozygous for susceptibility, H: marker allele heterozygous) recombinants in the QTL region. A: marker allele homozygous for resistance. Unique line names are indicated in the column on the left. The number of individual clones (*n*) tested per line is indicated in square brackets in the format of [STR4, TR4]. The marker names are displayed at the top, corresponding to the numeric part of the ‘DH-Pahang’ v1 gene accessions. Recombinations between adjacent markers are indicated by a solid vertical bar. One-way ANOVA probability (*p*) and eta-squared (η^2^) values are displayed at the bottom for each marker–phenotype comparison. Statistically significant comparisons at *p* < 0.05 for *Foc*-STR4 and *p* < 0.1 for *Foc*-TR4 are highlighted in bold. (**B**) *Foc*-STR4 phenotypes of the recombinants are scored as rhizome discolouration index (RDI). Red/blue bars indicate *Foc*-STR4-resistant/susceptible phenotypes, respectively. (**C**) *Foc*-TR4 sensitivity was scored as RDI in a subset of the critical recombinants. Disease incidence (grey) is indicated as a percentage of the number of individuals showing symptoms over the total number of clones (*n*) screened per genotype on a scale at the top. Asterisks (*) indicate that resistance was observed where a susceptible phenotype was expected. The respective +/− controls in the *Foc*-TR4 screening were the Cavendish cultivar Williams with or without the pathogen. RDI was scored according to a 1–8 scale [33] for *Foc*-STR4 and a 1–6 scale for *Foc*-TR4 [28]. The 95% confidence intervals of the means are plotted as error bars for lines with *n* > 2. Significant differences at *p* < 0.05 among groups were determined using one-way ANOVA. The means were separated by least significant difference at *p* ≤ 0.05. The subsets are indicated by letters in superscript.

Population 1 was used for genetic mapping. Eleven CAPS markers were developed to anchor the region underlying the STR4 QTL (Table 1). The most proximal (27960) and distal (30000) markers defined a 1.45 Mb region in ‘DH-Pahang’ v4 (Table 2). The markers are named according to their unique identifiers in ‘DH-Pahang’ v1, and their corresponding v4 gene models as well as their predicted proteins are listed (Table 2). The 11 co-dominant CAPS markers were mapped in 435 F_2_ individuals of Population 1. The genetic distance in centiMorgan (cM) was calculated as the number of progenies carrying a cross-over event between a pair of adjacent markers over the total number of individuals (Figure 2). Overall, the order of the genetic linkage map was consistent with the physical positions of these genes on chromosome 3 in ‘DH-Pahang’ v4, indicating the absence of large structural rearrangements in this region between the parental *M. acuminata* ssp. *malaccensis* lines and ‘DH-Pahang’ v4. A set of 32 lines carrying cross-over events in this region were phenotyped to further delimit this region (Figure 3A). Resistance was completely dominant over susceptibility at this locus. Therefore, only recombinants carrying a homozygous-B to heterozygous-H (B/H) or a H/B cross-over were tested. Recombinants carrying A/H or H/A cross-overs were not tested, as ‘A’ cannot be differentiated phenotypically from ‘H’. The recombinants were grouped according to their *Foc*-STR4 resistance and susceptibility (Figure 3B). In the *Foc*-STR4-resistant phenotypic group, the three *M. acuminata* ssp. *malaccensis* parents, ‘Ma850’, ‘Ma851’, and ‘Ma852’, along with nine recombinants, showed resistant phenotypes that were clearly separated from the susceptible progenies by least significant difference (LSD) (Figure 3B). Among them, the H/A recombinant line ‘18’ showed a resistant phenotype, but it is not informative for individuals carrying homozygous alleles for resistance (A), as it cannot be differentiated phenotypically from the heterozygotes (H). On the other hand, 23 recombinants showed *Foc*-STR4-susceptible phenotypes (Figure 3B). The susceptibility of these recombinants seemed to be highly elevated, with the majority of the clones exhibiting an RDI of 8 (plant death) by the time of harvest. The STR4 resistance locus is defined by three proximal recombinants (852-143, 852-168, and 4_16), with marker-phenotypes all suggesting that the locus is distal to marker 28420, and with four distal recombinants (852-7, 852-140, 852-162, and 81) collectively, suggesting that the locus is proximal to marker 29590 (Figure 3A,B). This defined the locus within a genetic interval of 12.9 cM between 28420 and 29590 (Figure 2). Furthermore, the marker phenotype of recombinant lines 194 and 852-108 indicated that the locus can potentially be refined to lie between markers 28820 and 29460 (Figure 3A,B), although additional recombinant lines are required to validate this interval. However, eta-squared (η^2^) values of marker–trait association are the highest at markers 28820 and 29460 (*p* = 0.05), confirming that they are positioned closest to the trait locus (Figure 3A).

TR4 phenotyping of a subset of critical recombinants produced a similar result (Figure 3C). The rhizome discolouration was scored on a scale of 1 to 6, with 1 corresponding to a healthy plant, and 2 through 6 corresponding to the proportion of discoloured rhizomes of ≤20%, ≤40%, ≤60%, ≤80%, and ≤100%, respectively. The phenotypic difference between the R and S recombinants were reduced in comparison with the STR4 phenotype (Figure 3C). The marker-defined susceptible lines were generally more resistant to TR4 than to STR4, with more clones per line that did not show any rhizome discolouration. The positive control ‘Williams’ showed an average RDI of greater than 60%, indicating that the inoculation method worked as intended. Separation of the means using Duncan’s multiple range test produced subsets that were more overlapping than those of STR4. Two S recombinants, 852-7 and 852-47, did not produce the expected symptoms, and their means were clustered together with the resistant recombinants and the uninoculated Williams (Figure 3C). This suggests that sensitivity to TR4 in *M. acuminata* ssp. *malaccensis* was not optimally detected at the current inoculum dosage. However, all susceptible recombinants except 852-7, 852-47, and 1 showed a disease incidence (number of plants that developed disease over the total number of clones (*n*) screened per genotype) between 20–100%. All critical recombinant phenotypes (except 852-7) were correctly associated with the direction of the trait locus between 28420 and 29590 (Figure 3A,C). The recombinants 194 and 852-108 also showed the expected association, with the closest flanking markers 28820 and 29460. Likewise, this region was also associated with the highest η^2^ values, at 0.17–0.18, *p* = 0.1 (Figure 3A). The phenotypic variation explained by TR4 at this locus was smaller than that controlled by STR4 (η^2^: 0.68–0.73).

### 2.3. Candidate R Gene Expression Profiling

A set of 24 Population 1 progenies that are homozygous for the resistant ‘A’ or susceptible ‘B’ for all eleven markers across this region were used to perform a transcriptome analysis with RNAseq. The phenotype of each of these lines was confirmed in a pot trial prior to the start of this experiment. The experiment was designed to identify a narrow transcriptome response that is specifically controlled by the resistance locus in this region. Genetic effects unlinked to this locus are accounted for by the segregation of these genes in the genetic background.

Our previous study identified multiple classes of R genes present in the candidate region [57]. Differential gene expression analysis was performed in a pairwise (R vs. S) manner at four time points, namely 0, 1, 3, and 7 days post-inoculation (dpi). Markers 28420 and 29590 flanked a 959 kb region containing 125 predicted gene models in ‘DH Pahang’ v4 (Table S1). Gene Ontology (GO) enrichment analysis of this region revealed two significantly enriched GO terms (*p*-adj. < 0.05) that were associated with plant defense under the ontology of ‘Biological Process’, namely ‘defense response to bacterium’ (GO:0042742, 7 genes) and ‘defense response to fungus’ (GO:0050832, 5 genes) (Table S2). Under ‘Molecular Function’, GO terms were significantly enriched for ‘polysaccharide binding’ (GO:0030247) and ‘endoribonucleae activity’ (*p*-adj. < 0.05).

**Figure 4:**
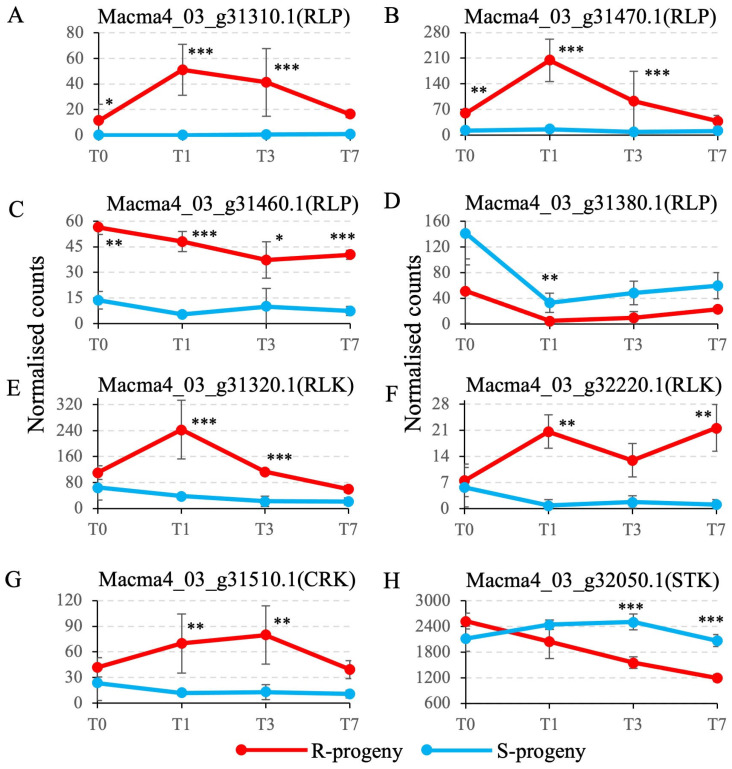
Differential expression (DE) of candidate genes. DESeq2-normalised gene counts using median-of-ratios method were calculated for DE genes selected from an RNAseq study of a seven-day *Foc*-STR4 infection time course using R- and S-progenies of Population 1. (**A**) Macma4_03_g31310.1, a putative LRR RLP protein. (**B**) Macma4_03_g31320.1, a putative LRR receptor-like serine/threonine-protein kinase. (**C**) Macma4_03_g31470.1, a putative LRR RLP protein. (**D**) Macma4_03_g31510.1, a putative cysteine-rich receptor-like protein kinase 6. (**E**) Macma4_03_g32220.1, a putative leaf rust 10 disease-resistance locus receptor-like protein kinase-like protein (LRK10L). (**F**) Macma4_03_g31460.1, a putative LRR RLP protein. (**G**) Macma4_03_g31380.1, a putative LRR RLP protein. (**H**) Macma4_03_g32050.1, a putative serine/threonine-protein kinase/endoribonuclease IRE1a. Replicates (*n*) per genotype per time point is 3. Significantly differential expression between R and S progenies was indicated at *p*-adj. < 0.05 (*), *p*-adj. < 0.01 (**), and *p*-adj. < 0.001 (***). T: time in days; RLP: receptor-like protein; RLK: receptor-like kinase; CRK: cysteine-rich kinase; STK: serine/threonine protein kinase. Error bars indicate standard errors of the means (*n* = 3).

Of all the R genes predicted in this region, seven genes showed differential expression profiles between R and S at two or more time points at *p*-adj. < 0.05 (Figure 4). Of the four receptor-like proteins (RLP), expression of 31310 and 31470 was upregulated at 1 and 3 dpi in R progenies before being downregulated at 7 dpi, although it remained relatively low in the S progenies throughout the time course (Figure 4A,B). Transcript levels of the RLP 31460 were significantly higher in R relative to S at all time points (*p*-adj. < 0.05) (Figure 4C). The transcript levels of 31460 steadily declined from 0 to 3 dpi in R but were maintained at a higher level in R than in S across all time points. In contrast, transcripts of the RLP 31380 were readily downregulated at 1 dpi before a slight recovery at 3 and 7 dpi in both S and R progenies and with R transcripts significantly higher (*p*-adj. < 0.01) than S transcripts at 1 dpi (Figure 4D). The receptor-like protein kinase (RLK) 31320 showed a similar profile to RLP 3130 and 31470 in that *Foc*-STR4 rapidly induced an expression peak at 1 dpi, followed by a gradual downregulation at 3 dpi before returning to a pre-treatment level at 7 dpi (Figure 4E). The 31320 transcripts in S genotypes were maintained at a low level throughout the experiment. Transcript levels of the other RLK gene 32220, a LRK10L homolog, were significantly upregulated at 1 dpi in R and were then upregulated further at 7 dpi (Figure 4F). Its transcripts in S remained relatively low at all time points. The cysteine-rich protein kinase (CRK) 31510 had an expression peak at 3 to 7 dpi in R before a sharp downregulation to a level comparable to the control at 7 dpi (Figure 4G). Again, the S transcripts were maintained at a relatively low level. Lastly, the serine/threonine protein kinase (STK) 32050 showed a strong downregulation in R across all time points (Figure 4H), whereas the S transcripts started at a similar level to R but were gradually upregulated at 1 to 3 dpi before returning to a pretreatment level at 7 dpi. No intracellular R proteins were differentially expressed at more than two time points between R and S in this region.

### 2.4. Foc-STR4 Resistance and Marker Validation in Population 2

**Figure 5:**
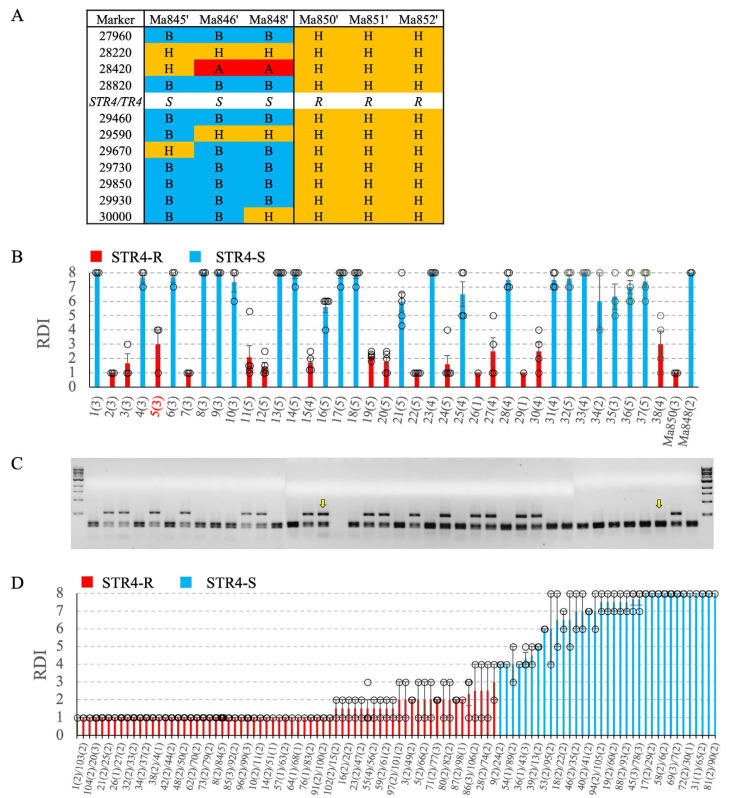
*Foc*-STR4 resistance and marker validation in the ‘Ma850’ × ‘Ma848’ population. (**A**) Marker haplotypes of the six parental *Musa acuminata* ssp. *malaccensis* in the QTL region. Marker allele annotations are described as in Figure 3A. The position of the *Foc*-STR4 and *Foc*-TR4 resistance locus is indicated. Parental *Foc* sensitivity, S: susceptible, R: resistant. (**B**) ‘Ma850’ × ‘Ma848’ F_1_ individuals screened with *Foc*-STR4. *Foc*-STR4-resistant and -susceptible phenotypes are differentiated by red/blue coded bars, respectively. RDI: rhizome discolouration index. The line (number 5) with red highlighting was used to generate the self-crossed F_2_ population. (**C**) A CAPS marker screening was performed on the ‘Ma850’ × ‘Ma848’ F_1_ individuals using the primers ‘28820-SNP8-F2’ and ‘28820-SNP8-R1’, targeting an SNP in gene model GSMUA_Achr3G28820 (‘DH-Pahang’ v1.0) and PCR conditions as described in Table 1. The dominant band (544 bp) after a BstZ17I digest is associated with *Foc*-STR4 resistance. Yellow arrows indicate de-coupling of the dominant marker band with *Foc*-STR4 resistance. (**D**) ‘Ma850’ × ‘Ma848’ F_2_ individuals screened with *Foc*-STR4. Individuals with an RDI score of < 4 are considered resistant (R), and those with an RDI score of ≥ 4 (greater than 20% discolouration) are considered susceptible (S). Individual x-axis labels are staggered every two lines. The number of clones (*n*) tested per line is indicated in brackets.

The haplotype analysis across the QTL region showed that the marker loci were all heterozygous in the R parents and were susceptible ‘B’ haplotype interrupted by heterozygous segments in the S parents (Figure 5A). The candidate region ‘B’ for susceptibility defined by 28820/29460 in the S parents was flanked by heterozygous segments at the proximal (28220–28420) and distal (29590–29670) ends (Figure 5A). Therefore, the marker haplotypes of the S parents were consistent with the location of the STR4/TR4 locus as defined by Population 1.

To validate the segregation of resistance observed in Population 1, 38 F_2_ progenies of the ‘Ma848’ × ‘Ma850’ cross were screened for STR4 resistance (Figure 5B). There were 16 R and 22 S phenotypes observed, while the parents, ‘Ma848’ and ‘Ma850’, showed the expected STR4 susceptibility and resistance, respectively. The mapping of 28820 in the F_1_ individuals showed that the dominant allele of 28820 closely segregated with resistance (Figure 5C). Decoupling of the marker with the trait occurred in F_2_ individuals ‘16’ and ‘34’, suggesting that recombination occurred between the resistance gene and the marker locus. An F_3_ population was developed using a self-cross of the STR4-resistant F_2_ individual ‘5‘. Of the 102 F_3_ individuals screened for STR4 resistance, 67 individuals were resistant (mean RDI < 4), and 35 individuals were susceptible (mean RDI ≥ 4) (Figure 5D), with goodness-of-fit statistics showing significant deviation from an expected segregation ratio of 3 R:1 S (χ^2^ = 4.71, *p* = 0.029, df = 1, α = 0.05).

### 2.5. Validation of Marker 29730 for Marker-Assisted Selection of TR4 and STR4

**Figure 6:**
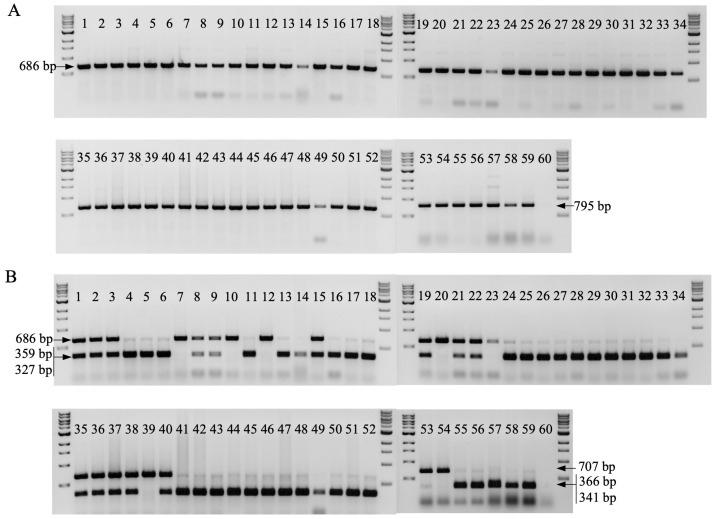
Marker validation for marker-assisted selection of *Foc* race 4 resistance in the diploid (AA) wild relatives and hybrids from the IITA collection. The SNP marker generated from GSMUA_Achr3G29730 in ‘DH-Pahang’ reference genome v1 was converted to amplify A-genome-specific products based on A/B genome discriminating SNPs at the 3’ termini of the primer pair (Table 1). (**A**) PCR amplification using 29730-SNP1-F1/29730-SNP1-R1 (Lane or L1-52, mostly diploids) and 29730-A-SNP1-F2/29730-A-SNP1-R2 (L53-60, mostly polyploids), respectively, amplified a single PCR product (686/795 bp) in 59 genotypes, as per Table 3. L60 is the *Musa balbisiana* (BB), which served as a negative control for the A-genome-specific PCRs. (**B**) This product was subsequently digested with BcoDI to reveal a dominant uncut band (686 bp/L1-52, 707 bp/ L53-60), putatively associated with resistance. The alternatively cut allele (359 bp and 327 bp/L1-52, 366 bp and 341 bp/L53-60) may indicate the presence of the *Foc*-susceptible allele. Accessions heterozygous for the marker locus were predicted to be resistant to *Foc*-STR4 and *Foc*-TR4 due to the complete dominance of the R allele over the S allele at this resistance locus. Resistances were detected in ‘Ma850’ (L1), ‘Ma851’ (L2), ‘Ma852’ (L3), ‘Pahang’ (L7, 20), ‘SH-3362’ (L8, 9, 37), ‘Madang Gaudelope’ (L10), ‘SH-3217’ (L12), ‘Malaccensis-ITC0250’ (15, 38), ‘Malaccensis-ITC0399’ (L19), ‘Pa Musore no2’ (L21), ‘Kluai Pal’ (L22), ‘CIRAD 930/DH Pahang’ (L23), ‘TMB2X7197-2’ (L35), ‘5610S-1’ (L36), ‘SH-3217’ (L39), ‘SH-3361’ (L40), ‘FHIA 3’ (L53), and ‘FHIA 25’ (L54). Resistances were not detected in other known *Foc*-resistant *M. acuminata* ssp., such as *M. acuminata* ssp. *burmannica* ‘Calcutta 4’ accessions (L11, 13), or in cultivated diploid AA varieties, such as ‘Pisang Jari Buaya’ (L14) and ‘cv. Rose’ (L44). A 1kb DNA ladder from NEB was used as a reference for the size of the amplicons.

To identify SNPs that may be used in detecting the resistance locus outside of our mapping population, we first interrogated the SNPs in the CAPS markers for their association with resistance in a small set of accessions (namely all our *Musa acuminata* ssp. *malaccensis* parents, ‘DH-Pahang’, ‘Pahang’, ‘SH3362’, ‘FHIA25’, ‘Pisang Jari Buaya’, and ‘Calcutta 4’) that are known to carry STR4/TR4 resistance. Of all the markers tested, only one marker, 29730, showed an association with STR4/TR4 resistance in a subset of these genotypes. All the other SNPs interrogated were not correlated with the resistance/susceptibility of accessions outside of the mapping populations. This marker, along with A-genome (*M. acuminata*)-specific primers for 29730-A were subsequently developed (Table 1) and used to amplify a single PCR product of 686/795 bp (29730/29730A) in a set of 60 banana wild and cultivated accessions (Figure 6A). This product was then digested with BcoDI to produce the bi-allelic forms (an undigested dominant band that is putatively associated with resistance) and digested products linked to susceptibility (Figure 6B). Heterozygotes carried both variants. The dominant marker allele was detected in the parents, ‘Ma850’, ‘Ma851’, and ‘Ma852’, and six other *Musa acuminata* ssp. *malaccensis* accessions, ‘Pahang’, ‘CIRAD 930/DH Pahang’, ‘Malaccensis ITC250’, ‘Malaccensis ITC0399’, ‘Pa Musore no2’, and ‘Kluai Pal’ (Figure 6B, Table 3). Hybrids and cultivars that had the resistant band include ‘SH3361’, ‘SH3362’, ‘SH3217’, ‘TMB2×7197-2’, ‘5610S-1’, ‘FHIA3’, and ‘FHIA25’. Other known resistant lines, such as ‘cv. Rose’, ‘SH-3142’, ‘IV9 Calcutta4’, ‘Pisang Jari Buaya’, as well as the negative control M. balbisiana, did not produce the dominant band (Figure 6B, Table 3). This suggests that the resistance source was prevalent among *M. acuminata* ssp. *malaccensis* and its derivatives. Its absence in ‘cv. Rose’, a *M. acuminata* ssp. *malaccensis* known to be resistant to TR4, and other TR4 resistant lines that are not of *M. acuminata* ssp. *malaccensis* origin suggests the presence of resistance sources elsewhere in the genome.

To further test this marker and aid the marker-assisted selection of *Foc*-STR4- and *Foc*-TR4-resistant lines, we screened 72 accessions from the IITA collection (Uganda) and 46 accessions from the IITA’s *M. acuminata* ssp. *banksii* collection (Nigeria). Of the 11 ‘Matooke’ tetraploid parents screened, two of them, ‘1438K-1’ and ‘376K-7’, were positive for the resistant band (Table S3). Of all the ‘NARITA’ triploids that were assessed for yield stability in Uganda and Tanzania [63], line numbers 1, 5, 6, 7, 8, 9, 13, 15, 16, 17, 19, 22, 23, and 25 carried the dominant allele. In the ‘NARITA’ triploids and the other triploid hybrids screened, the presence of the dominant allele in the heterozygous state (H) was most likely inherited from their male diploid parents, namely ‘SH3362’, ‘5610S-1’, ‘TMB2 × 7197-2’, ‘SH3217’, and ‘Malaccensis_250’ (Table S3). Heterozygotes were detected in 6 out of the 18 hybrid triploids that used ‘Malaccensis_250’ as the male parent. This is consistent with the heterozygous genotype of ‘Malaccensis_250’ at this locus. The screening of 46 accessions from a cultivated and wild *M. acuminata* ssp. *banksii* collection did not detect the dominant allele, with the positive control being ‘SH3362’ (Table S4).

## 3. Discussion

Conventional breeding is typically constrained in banana because polyploid cultivars are sterile and parthenocarpic [64]. Development of large segregating populations can be achieved using highly fertile banana diploids. The underlying genetics in banana are still challenging due to their long growth cycles, the logistics of performing high-throughput screenings, and the high variability in the phenotypic data, as reflected in this study. Despite these difficulties, the availability of the *Musa* draft genome assemblies and lower whole genome genotyping/sequencing costs have facilitated studies in SNP discovery, genome evolution, and population genetics in banana [65,66,67,68,69]. With *Foc*-TR4 edging closer to the major banana growing regions of Latin America [70], it becomes ever more important to dissect host resistance against *Foc*-TR4 and, in doing so, to identify potential resistance genes that underpin the *Foc*-TR4 resistance per se. This would allow resistance to be deployed in elite cultivars by gene editing or through a transgenic approach. Molecular markers that are closely linked to TR4-resistant QTLs can fast-track resistant alleles in banana-breeding programs.

By using transcriptome sequencing on S or R progenies carrying contrasting haplotypes in the QTL region, candidate R genes underlying resistance were identified. Segregant analysis is a powerful approach when combined with the positional information from genetic mapping. Firstly, the candidate region was confirmed in Population 1. The marker haplotype in the susceptible parents and the segregation of *Foc*-STR4 further independently confirmed the candidate region in Population 2. The closely linked marker 28820 segregated with STR4 resistance, although not completely, but the phenotypic variation explained at marker loci 28820 and 29460 was the highest in this genetic interval for both STR4 and TR4. Within this region, 32220, a leaf rust 10 disease-resistance locus receptor-like protein kinase-like protein 2.1 (LRK10L-2.1) was related to the wheat LRK10 gene [71]. Transcripts of 32220 were gradually and consistently upregulated in R progenies during the time course, peaking at 7 dpi. This response was not detected in the S progenies. The 32220 predicted protein belongs to the LRK10L-2 subfamily of receptor-like kinases [72,73] and has a cysteine-rich ectodomain, a transmembrane domain, and a predicted intracellular serine/threonine kinase at its C-terminus. Members of this class of RLKs have been shown to be important for mediating resistance responses to stripe rust fungus and powdery mildew in wheat [74,75], and they are involved in ABA-mediated signaling and drought resistance in Arabidopsis [76].

The genetic Interval closest to the STR4 resistance locus is between 28820 and 29590. It is not well-defined at this stage. Only two individuals were identified with crossovers between these markers. More recombinants are needed to narrow this interval more precisely. In the larger region between markers 28840 and 29590, multiple recombinants consistently confirmed the direction of the trait locus on either side. Although one critical recombinant (852-7) did not produce any symptoms in the TR4 screening, the phenotypic data were generally concordant with the genetic interval defined for both STR4- and TR4-resistant loci. Within this interval, there was a cluster of receptor-like kinases (LRR XII subfamily) and receptor-like proteins (LRR RLP subfamily) positioned in an interspersed arrangement [57]. They, respectively, belong to the LRR XII and LRR RLP subfamilies of pattern recognition receptors [72,77]. Two RLPs showed a very rapid upregulation of transcripts at 1 dpi, consistent with their roles in the recognition of pathogen effectors at the onset of infection [78]. These RLPs are similar to the tomato LeEIX1 and LeEIX2 resistance proteins that directly interact with an ethylene-inducible xylanase (Eix) effector protein from *Trichoderma viride* [79]. Similarly, an Eix-like effector (VdEIX3) from *Verticillium dahlia* was recognised by the *Nicotiana benthamiana* LRR RLP NbEIX2 [80], inducing an innate immunity response and increasing the resistance to other oomycete and fungal pathogens in *N. benthamiana*.

A gene encoding a cysteine-rich protein kinase was also strongly upregulated during the onset of infection in the R but not in the S genotypes. Cysteine-rich protein kinases contained DUF domains and a kinase domain. Such genes have been found to confer resistance against *Septoria tritici* blotch and leaf rust in wheat [81,82]. Overexpression of an Arabidopsis CRK homolog led to enhanced resistance against *Pseudomonas syringae* [83]. In addition, an LRR RK gene (Macma4_03_g31320.1) was differentially expressed between the S and R genotypes and exhibited an expression peak at 1 dpi in R, similar to the profiles of the three LRR RLPs. Plants, in general, have an abundant amount of RLKs and RLPs as part of their surveillance system to cope with the evolution and detection of pathogens [84]. The LRR ectodomain of pattern recognition receptors binds to proteins and peptides through pathogen-associated molecular patterns (PAMPs) or damage-associated molecular patterns (DAMNs) and is important for the recognition function. In Arabidopsis, FLAGELLIN SENSING2 (FLS2) recognises an elicitor epitope from the bacterial flagellin [85], and PEP RECEPTOR 1 (PEPR1) and PEPR2 recognise plant elicitor peptides, or peps, to activate a defense against *Pythium irregulare* [86,87]. In rice, LRR RK Xa21 recognises a highly conserved protein, RaxX, from *Xanthomonas* species to trigger immune responses [88].

Overall, there are multiple resistance genes differentially expressed between the S and R banana progenies with similar temporal expression profiles. All of them are indicative of a rapid response in the induction of resistance gene transcripts at the onset of STR4 infection. This suggests that these genes may act in close proximity to one another or even belong to the same gene network. Co-expression gene networks will be constructed from RNA sequencing data to identify co-expression modules. This information can then be integrated with the QTL region to characterize the candidate genes [89].

In this study, we demonstrated that SNP loci/trait associations can produce markers useful for marker-assisted selection. Unlike traditional bi-parental mapping, the wild subspecies of *Musa* are highly heterozygous, which render it challenging for genetics to be undertaken. The resistance source identified in this population was dominant, which is consistent with the mode of inheritance of a race 1 and, to a lesser extent, TR4-resistant QTLs located on chromosome 10 of a different *Musa acuminata* ssp. *malaccensis* [54]. The dominance of these loci can offer full TR4 protection, which is a desirable genetic solution to the TR4 pandemic since only one copy of the gene(s) is required to confer full resistance against TR4/STR4. Resistances that are not completely dominant may not be useful since partial resistance cannot offer protection against TR4 in the long term [90].

In marker-assisted selection, we used a marker closely linked to the resistance locus to detect lines potentially carrying this locus from several germplasm collections. Initial screening clearly suggested that this marker could identify some of the resistant individuals in the diploid collection, specifically detecting resistance in wild relatives or derivatives of *M. acuminata* ssp. *malaccensis* origin (Figure 6, Table 3). The power of detection did not extend to other *M. acuminata* subspecies or derivatives that were not of *M. acuminata* ssp. *malaccensis* origin. This was evident in that this marker failed to detect resistance in the *M. acuminata* ssp. *banksii* collection (Table S4). Furthermore, the *M. acuminata* ssp. *burmannica* genotype ‘Calcutta 4’ has been reported to be highly resistant not only to STR4/TR4 [33,43] but also to the Sigatoka leaf spot disease [91]. ‘Calcutta 4’, as a source of resistance, has already been used extensively in IITA-NARO’s breeding program. It was used as a male parent to derive seven tetraploid ‘Matooke’ hybrids, which were used to derive the triploid ‘Matooke’ NARITAs [92,93] (Table S3). Despite being TR4-resistant, ‘Calcutta 4’ was not detected as resistant in the marker screening in our study. Taken together, this highlights the presence of other sources of resistance in the germplasm collection as well as the limitation of this marker to detect resistance sources outside of *M. acuminata* ssp. *malaccensis*, possibly reflecting the phylogenetic divergence of the *M. acuminata* subspecies in the core *Musa* collection [59]. Overall, the marker was positive in 35 of 72 individuals in the IITA collection, exhibiting a detection frequency of 47.9%. This predicted that the chromosome 3 resistance source was already present in the IITA-NARO’s breeding program.

The genotype screen also produced consistent results in the diploids, specifically ‘Pahang’, ‘DH-Pahang’, and ‘Malaccensis-ITC0250’. These are known TR4/STR4-resistant genotypes. In the hybrids, ‘SH3362’ and ‘SH3217’, are positives for the dominant band. ‘SH3362’ was derived from crossing ‘SH3217’ and ‘SH3142’, with the latter derived from a cross between two cultivars of ‘Pisang Jari Buaya‘ ‘https://www.promusa.org/NARITA+16’ (accessed on 12 March 2023). Despite being resistant to TR4, ‘Pisang Jari Buaya’ was a negative in our marker screen. The parentage of ‘SH3217’ can be further traced back to a cross between ‘SH2095’ and ‘SH2766’. ‘SH2095’ was derived from a cross between ‘Sinwobogi’ (AA) and ‘Tjau Lagada’ (AA), whereas ‘SH2766’ was derived from ‘Tjau Lagada’ (AA) and the progeny of a cross between *M. acuminata* ssp. *malaccensis* and ‘Guyod’ (AA) ‘https://www.promusa.org/NARITA+16’ (accessed on 12 March 2023). Therefore, the source of resistance potentially can be traced back to a *M. acuminata* ssp. *malaccensis* origin, although validation is not possible without these progenitors or their DNA. ‘SH3362’ and its progenitor ‘SH3217’ were the male parents of 13 hybrids in the IITA collection (Table S3). Ten of these thirteen hybrids were heterozygous for the STR4/TR4 marker locus. Despite the common presence of this resistance source in the IITA-NARO’s breeding program, further phenotypic screening in the IITA germplasm is required to validate this marker. Breeding programs around the world can now use this as a tool to identify potential TR4-resistant genotypes in their collections. This is a first-ever report on PCR-based marker-assisted selection in a banana-breeding program. It will assist efforts towards curbing the TR4 pandemic.

The genetic mapping using 435 individuals of Population 1 delimited the QTL to a 959 kb region containing 125 predicted gene models between 28420 and 29590 in ‘DH Pahang’ v4 (Table S1). Due to the sheer volume of the population and the number of clones that would have to be multiplied in vitro, phenotyping the entire population was never the goal. A targeted strategy was used to define the QTL region, and only recombinants were tested. It allowed ‘walking’ along the chromosome to define the direction of the marker–trait association. Validation was achieved through testing multiple independent recombinants defining a single marker interval. Technical bottlenecks included slow multiplication of clones in the diploid (AA) lines, as they sometimes have reduced shoot proliferation potentials compared with the triploids. Furthermore, the dominant mode of inheritance means that phenotypic distinction can be made only between H/A and B and vice versa. Individuals containing cross-over events between A and H marker alleles cannot be used unless progeny testing is performed at the next generation. Important A/H recombinants can be tested this way, although it is a labor-intensive task.

Given that it takes 3 months for sufficient clones to be multiplied, 1 month for the plants to be hardened off in a glasshouse, and an additional 3 months post-inoculation for symptoms to develop, this type of screening where genotypes are consistently processed in batches in an optimized and high-throughput manner is just not achievable with field-based trials. Future work will focus on optimizing high-throughput setups in glasshouses [94] or growth chambers where relatively young plants in small pots and trays can be screened with *Foc*. Screening in a controlled environment can reduce variance in the symptoms. Lab-based soil-free hydroponic systems have been explored for TR4 screening [95,96] and have been used to assay Fusarium root rot in other plant species, such as alfalfa [97]. High-throughput screening methodology from other plant/Fusarium pathosystems, such as *Medicago truncatula*/*F. oxysporum* f. sp. *medicaginis*, can potentially be adopted to screen for TR4 resistance in banana seedlings [98].

The STR4 screening produced clear-cut phenotypic differences between resistant and susceptible individuals. A hybrid inoculation method was used with spore suspension and an extra layer of millet added on top of the soil. This was implemented to increase the inoculum dosage and achieve uniformity with the infection. This allowed genotypic sensitivity to *Foc* to be detected reliably and the genetic interval to be defined. The TR4 screening also produced consistent results and identified the same genetic interval, although the plants, in general, did not produce symptoms as severe as STR4. The TR4 symptoms were slow to manifest, indicating that *M. acuminata ssp. malaccensis* were generally more resistant to TR4 than to STR4 in pot trials. The weaker correlation could be due to the presence of the chromosome 10 QTL for TR4 resistance in a fixed state in our resistant parents [54], which may also explain the segregation distortion we observed in the analysis of the F_3_ progenies from Population 2. Image-based detection of symptoms can assist in the quantification of rhizome discolouration [40]. The issue with the TR4 screening was not the subtle differences in the level of discolouration but rather obtaining false negatives when symptoms were expected. Symptom severity was able to be elevated by an increase in the inoculum dosage. That, in turn, reduced the variance in the symptom development. Overall, this highlights the challenge of detecting a plant’s sensitivity to *Foc* in a reliable manner.

## 4. Materials and Methods

### 4.1. Musa acuminata ssp. Malaccensis Populations

Three *Foc* race 4-resistant and three susceptible *M. acuminata ssp*. *malaccensis* parents were used in this study. The progenies of the R (resistant) parents ‘Ma850’, ‘Ma851’, and ‘Ma852’ segregated for *Foc*-STR4 and *Foc*-TR4 resistance [57,58], whereas the S (susceptible) parents ‘Ma845’, ‘Ma846’, and ‘Ma848’ were uniformly susceptible to *Foc*-STR4 (Figure 1A). Three close-pollinated F_2_ populations, collectively called Population 1 and consisting of 435 individuals, were developed for mapping. They consisted of two self-crosses of ‘Ma851’ and ‘Ma852’ as well as an inter-cross between these two lines (Figure 1B). Segregation of STR4 resistance was further validated in Population 2 (38 F_2_ and 102 F_2_ individuals), which was derived from an inter-cross between ‘Ma850’ and ‘Ma848’.

### 4.2. Fungal Isolates

For the *Foc*-STR4 screening, three monoconidial VCG0120 isolates (BRIP63488, BRIP43781, and BRIP42331) from the Queensland Plant Pathology Herbarium were used as a combined inoculum at the University of Queensland. For the *Foc*-TR4 screening, a VCG01213/16 isolate from the culture collection of Stellenbosch University’s Department of Plant Pathology was used.

### 4.3. Foc-STR4 Pot Trial

*Foc*-STR4 pot trials were conducted in temperature-controlled glasshouses at the University of Queensland, St Lucia campus, QLD, Australia. The temperatures were controlled at 26 °C day/22 °C night for the entire duration of the experiments. Humidity was maintained at 60%. The amount of 50mL of 2.0 × 10^6^ conidia/mL solution was poured directly into potted plants with a stem height of 30 cm, followed by spreading a layer of *Foc*-STR4-infested millet (20–30 g) on the surface of the soil. Protocols for preparing *Foc*-infested millet and conidia suspensions were previously described [33,99]. The soil surface was then topped with a thin layer of potting mix. The plants were watered lightly. Internal disease symptoms were scored 3 months post-inoculation. A 1–8 rhizome scale was used to score internal rhizome discolouration [33].

### 4.4. Foc-TR4 Pot Trial

*Foc*-TR4 pot trials were performed in a quarantined glasshouse at the University of Stellenbosch. Plants were hardened off for 2–3 months before the screening. The experimental setup for the pot trial was as previously described [100]. A millet inoculation technique was used, and disease incidences and internal discolouration of the rhizome (1–6 scale) were scored as per a previous study [101]. The positive and negative controls were uninoculated and *Foc*-TR4-inoculated Williams, respectively.

### 4.5. Molecular Marker Development

SNPs were initially identified using a sequencing approach. The 100 bp paired-end sequencing was performed using the Illumina Genome Analyzer IIx platform (Illumina, San Diego, CA, USA) at the Australian Genome Research Facility, VIC, Australia, to produce 10x coverage for individually sequenced S and R libraries. There were 6 S libraries prepared, consisting of each of the 3 susceptible parents, ‘Ma845’, ‘Ma846’, and ‘Ma848’, as well as a pool of 34 susceptible progenies of ‘Ma845’, a pool of 3 susceptible progenies of ‘Ma851’, and a pool of 8 susceptible progenies of ‘Ma852’. Six R libraries were prepared. They consisted of each of the 3 resistant parents, ‘Ma850’, ‘Ma851’, and ‘Ma852’, and 3 DNA pools of 11, 17, and 24 resistant progenies (either homozygous or heterozygous for resistance), respectively, derived from ‘Ma850’, ‘Ma851’, and ‘Ma852’. Data generated from individual libraries were used to align to ‘DH Pahang’ v1 using SOAPaligner v2.21 [102], and SNPs were called using SGSautoSNP (Second-Generation Sequencing AutoSNP) [103]. SNP profiles were visualised in an aligned format using the Integrative Genomics Viewer [104], and gene models from ‘DH-Pahang’ v1 ‘https://banana-genome-hub.southgreen.fr’ (accessed on 5 March 2023) were used to identify genes and SNPs suitable for marker development. Restriction enzyme-cutting sites covering the SNP site were identified using ‘NEB cutter v2.0’ ‘https://nc2.neb.com/NEBcutter2/’ (accessed on 5 March 2023). Enzymes that had multiple restriction sites within a 400 bp region flanking the SNP on each side were avoided. Primers flanking a 344–795 bp amplicon were designed using ‘Primer 3’ [105] and further checked for binding specificity using ‘Oligoanalyzer’ ‘https://sg.idtdna.com/calc/analyzer’ (accessed on 5 March 2023).

### 4.6. DNA Extraction and PCR

DNA extraction was performed using a hexadecyltrimethylammonium bromide (CTAB)-based method [106], with modifications as follows: At the washing step, the DNA pellet was washed three times with 8 mL of 70% ethanol to reduce residual salt contaminants and finally resuspended in 400 μL of nuclease free water. The DNA was quantified on a NanoDrop UV/Visible spectrophotometer for a single absorbance peak at 260 nm, with a 260 nm/280 nm absorbance ratio of 1.8 to 2.0. DNA was then checked using the broad-range Bradford assay on a Qubit machine and finally visualised on a 0.7% (*w*/*v*) agarose gel to check for band shearing and/or contamination with either RNA or polysaccharide.

PCR was performed using 80–100 ng of DNA template and Dreamtaq (Thermo Fisher Scientific, Waltham, MA, USA). Running conditions were set according to the manufacturer’s recommendations. The primers and the corresponding annealing temperatures were optimized (Table 1). Forty cycles of PCR were used per reaction. Restriction enzyme digest was performed on 10 μL PCR product and 2 μL enzymatic mix consisting of 2 units of the enzyme and an appropriate 10× buffer (Table 1). The digested products were visualised on a 2% agarose gel with a 1 Kb ladder (New England Biolabs, MA, USA). The markers were scored in a co-dominant manner, with restriction band patterns differentiating one homozygous allele from the other. The heterozygotes contained both allelic forms.

### 4.7. Digital Gene Expression Analysis on Candidate Genes

A transcriptome study was performed by using 12 R and 12 S progenies from Population 1. These progenies were tested against STR4, and their resistance/susceptible phenotypes were confirmed prior to the start of this experiment. A root-dipping method using *Foc* spore suspension was used to inoculate the plants [33], and whole roots in triplicates (*n* = 3) were harvested at 0, 1, 3, and 7 days post-inoculation (dpi). Samples were snap-frozen in liquid nitrogen and then ground to powder using a mortar and pestle. Spectrum^TM^ Plant Total RNA kit (Sigma-Aldrich, MO, USA) was used to extract RNA. Here, 24 cDNA libraries corresponding to the R and S progenies harvested at the 4 time points were prepared and then sequenced using the Hiseq 4000 platform (Genewiz, Suzhou, China), generating approximately 48 Mb of 150 bp paired-end reads for each sample. Adaptor sequences and low-quality reads were filtered out using ‘Fastp’ [107]. Clean paired-end reads were then aligned to ‘DH-Pahang’ v4 reference genome using ‘STAR’ v2.7.10a and default parameters for all except ‘-outFilterMismatchNmax 6’ and ‘-alignIntronMax 10000’ [108]. Non-normalized read counts were tabulated with ‘FeatureCounts’ software (option: -M -g ID -t gene -p) [109] and then normalised to account for differences in sequencing depth among samples using the median-of-ratios method [110]. This value was calculated as the gene counts divided by a size factor specific to a sample, determined by the median ratio of gene counts relative to geometric mean of the gene counts per gene. DEGs were identified from pairwise comparisons between resistant and susceptible progenies at each time point using the ‘DESeq2’ R package [111]. Multiple testing was corrected using the Benjamini and Hochberg method [112]. The *p*-values were adjusted (*p*-adj.) to have a false discovery rate (FDR) cut-off of 0.05.

### 4.8. Statistical Analyses

The statistical software SPSS v28.0.1.0 (142) (IBM Corp., Armonk, NY, USA) was used to perform the statistical analysis described in this study. One-way ANOVA was performed in a pair-wise manner, with phenotype set as a dependent variable and marker-defined genotypes (B/H) as factors, to compare the means of STR4 and TR4 sensitivity at these loci. Any ‘A’ alleles were considered as ‘H’ for the purpose of statistical analysis, as resistance is completely dominant over susceptibility at this locus. The eta-squared (η^2^) values on the phenotype were estimated on the basis of the fixed-effects model and reflected the phenotypic variation explained at each marker-defined locus. To analyze the STR4 and TR4 phenotypes of the recombinants, Waller–Duncan’s multiple range testing was performed as a post hoc test to separate the means of the recombinants into subsets by least significant difference (LSD). Recombinants with *n* < 2 were excluded from the analysis. The harmonic mean sample size was estimated and used to account for the unequal variances associated with the uneven sample sizes (*n*) of the recombinants. The type 1/type 2 error seriousness ratio (k-ratio) was set to 100 (α = 0.05).

## 5. Conclusions

This study is the first-ever report of marker-assisted selection of STR4- and TR4-resistant *Musa* accessions. The availability of molecular makers closely linked to the resistance locus can now facilitate the rapid screening of potentially TR4-resistant genotypes and thereby reduce the generation time required for phenotypic and field trials. However, this marker can detect resistances originating from *M. acuminata* ssp. *malaccensis* at this locus only. Given the prevalence of TR4 now threatening the entire banana industry worldwide, identification of candidate receptors, such as proteins and kinases with strong transcriptional evidence linking them to resistance at this locus, provides the first step towards molecular dissection of resistance mediated by these R genes in banana.

## Figures and Tables

**Table 1:** CAPS marker information. The numeric identifier in primer names corresponds to the gene models of ‘DH Pahang’ assembly v1 without the prefix ‘GSMUA_Achr3G’. T is the annealing temperature used in the PCR. Frag or fragment denotes the PCR amplicon size in base pairs (bp). In the ‘Cut sizes’ column, lengths of the digested products are shown for the R and S marker alleles. Superscript ‘m’ indicates a monomorphic SNP cutting site. The SNP position (R to S nucleotide change) is calculated from the predicted translation start site AUG or ‘ATG’ in the genomic sequence of ‘DH-Pahang’ v4 gene models (SNP^ATG^).

Primer Name	Primer Sequence (5′ to 3′)	T (°C)	Frag (bp)	Cut by	Cut Sizes (bp)	SNP^ATG^
27960-SNP1-F1	GACCAGCAGCAGAAGGACCAGACC	58	764	BsaI	R:764	Exon1
27960-SNP1-R1	AGAATGAGTGGTATGGGAT				S:394,370	T1152C
28220-SNP8-F1	CCTGATTGTAAATGGGAAGTTTCTC	56	546	MnlI	R:292,223,31^m^	Intron2
28220-SNP8-R1	ATCGCCCAGCAGTGATTTGA				S:515,31^m^	G3100A
28420-SNP1-F1	CAAATATGCTGCTCCATCTG	54	740	NsiI	R:740	Intron4
28420-SNP1-R1	CTTGGAAGAAACTAACGAGTGT				S:403,337	A2547G
28820-SNP8-F2	CAGGTAACCATTTAGACTGACAA	55	544	BstZ17I	R:544	Exon3
28820-SNP8-R1	AATCAAGGAAATAGGGTGGCAC				S:300,244	C3274T
29460-SNP21-F2	GGATACTTGGACCCTGAGTACCAT	58	344	XhoI	R:313,31^m^	Exon4
29460-SNP21-R1	CCATCGCTCTCTATTGCTTGC				S:178,135,31^m^	T6353C
29590-SNP1-F1	GCTCAGATGTCTCAGTCCAGA	55	457	BstNI	R:457	Exon1
29590-SNP1-R1	CTTCTTCCATCCTCTTCTCC				S:317,140	A137G
29670-SNP8-F1	AAGAGATGTCATGTTGGTTCATTTG	56	628	BspCNI	R:628	Intron5
29670-SNP8-R1	CACTCACTCCTGCTATGCGGTTG				S:345,283	G5078C
29730-SNP1-F1	ATGGCACAGGTGATGTCAGT	58	686	BcoDI	R:686	Intron1
29730-SNP1-R1	ACTAGATGACTCAGATTAGTAGG				S:359,327	T544C
29730-A-SNP1-F2	GCAATGAGTACCTCTAAGCA	52	795	BcoDI	R:707,88^m^	Intron1
29730-A-SNP1-R2	TAAGTTCTAGTATCAAGTACAA				S:366,341,88^m^	T544C
29850-SNP13-F2	CTTGTTCCTGTTACCTATTAG	56	363	StyI	R:363	Intron5
29850-SNP13-R1	CCTTGTGCCTAGATGCTTGG				S:192,171	A4287G
29930-SNP1-F2	GTTCACACCCTTGACATCCTA	54	493	MseI	R:190,64,99^m^,49^m^,36^m^,30^m^,25^m^	Intron4
29930-SNP1-R1	TAAGCATTCATTAGCAAACGG				S:254,99^m^,49^m^,36^m^,30^m^,25^m^	A3401G
30000-SNP2-F2	CTTAAAACTTGGCGGAAGG	56	468	NsiI	R:251,217	Exon14
30000-SNP2-R2	CTGAAGCACAACTGTCCTTG				S:468	A6749G

**Table 2:** The ‘DH-Pahang’ reference genome v1 and v4 gene models for the CAPS markers developed in this study. The prefix of the v1 and v4 gene models are shown in brackets. The coordinates of the gene models defined on chromosome 3 of the ‘DH-Pahang’ v4 are shown in base pair (bp) ‘https://banana-genome-hub.southgreen.fr/’ (accessed on 23 February 2023). A plus (+) or minus (−) symbol indicates the positive and negative DNA strand designation, respectively, in the reference genome with respect to the transcriptional start of the gene models.

‘DH-Pahang’ v1 (GSMUA_Achr3G)	‘DH-Pahang’ v4 (Macma4_03_g)	‘DH-Pahang’ v4 Position (bp)	Description
27,960	30,750	40,893,205—40,895,172 (−)	MHD domain-containing protein
28,220	31,030	41,068,780—41,075,115 (−)	Uncharacterized membrane protein At1g16860-like
28,420	31,200	41,183,294—41,197,461 (−)	F-box domain-containing protein
28,820	31,680	41,695,490—41,699,989 (+)	Bifunctional nuclease 2
29,460	32,270	42,052,018—42,058,909 (+)	Leaf rust 10 disease-resistance locus receptor-like protein kinase-like 1.3
29,590	32,440	42,138,268—42,142,592 (−)	Pentatricopeptide repeat-containing protein At4g28010
29,670	32,510	42,186,029—42,193,520 (−)	Cycloartenol-C-24-methyltransferase 1
29,730	32,560	42,210,035—42,215,274 (−)	Nuclear transcription factor Y subunit A-1
29,850	32,690	42,283,482—42,289,346 (+)	WRKY transcription factor SUSIBA2
29,930	32,770	42,323,762—42,327,884 (−)	Hypothetical protein
30,000	32,830	42,349,497—42,357,604 (−)	Long chain base biosynthesis protein 2d

**Table 3:** Validation of marker 29730 for marker-assisted selection. Collection of diploids, improved diploids, cultivated diploids, and synthetic polyploids screened for the A-genome-specific marker 29730 (GSMUA_Achr3G29730) linked to both *Foc*-STR4 and *Foc*-TR4 resistance on chromosome 3 of *M. acuminata* ssp. *malaccensis* (Figure 6). The subspecies of *M. acuminata* or genome group is indicated in brackets next to the names. Het: heterozygous for the marker locus. Samples that form part of a collection are annotated as the following: ^a^ Diploid and cultivated varieties and ^d^ polyploid varieties from the Maroochy Research Facility, Department of Agriculture and Fisheries, Nambour, Queensland, Australia; ^b^ *M. acuminata* ssp. *malaccensis* accessions that form part of the core *Musa* collection used in a diversity study [59]; ^c^ improved diploids and a selected number of breeding lines from IITA, Uganda. *Musa balbisiana* (BB genome) served as a negative control for A-genome-specific amplification of 29730. In the *Foc*-STR4 and *Foc*-TR4 columns, resistances were generally defined as R: resistant, SS: slightly susceptible, S: susceptible, and n/a: data not available. Phenotypic data was referenced from multiple studies performed as either pot or field trials. ITC numbers are indicated on accessions where available, while other numbers correspond to accessions in their respective germplasm collections (MMC—NARO, Uganda; MRF—Maroochy Research Facility, QLD, AUS; and MUSA—INIVIT, Cuba).

Line	Name (Subspecies/Genome)	Accession	29730 Marker Locus	*Foc*-STR4	*Foc*-TR4
1 ^a^	‘Ma850’ (malaccensis)	MRF850	+(Het)	R [33]	R [33,58]
2 ^a^	‘Ma851’ (malaccensis)	MRF851	+(Het)	R [33]	R [58]
3 ^a^	‘Ma852’ (malaccensis)	MRF852	+(Het)	R [33]	R [58]
4 ^a^	‘Ma845’ (malaccensis)	MRF845	-	n/a	n/a
5 ^a^	‘Ma846’ (malaccensis)	MRF846	-	S [33]	n/a
6 ^a^	‘Ma848’ (malaccensis)	MRF848	-	S [33]	S [33,58]
7 ^a^	‘Pahang’ (malaccensis)	MRF1649	+	R [33]	R [33,45]
8 ^a^	‘SH-3362’ (AA)	MRF2010	+(Het)	R [33]	R [33,43]
9 ^a^	‘SH-3362’ (AA)	MRF2013	+(Het)	R [33]	R [33,43]
10 ^a^	‘Madang Guadeloupe’(malaccensis)	MRF655	+	R [33]	R [33]
11 ^a^	‘Calcutta 4’ (burmannica)	MRF1642	-	R [33]	R [33,45]
12 ^a^	‘SH-3217’ (AA)	MRF2005	+	R [33]	R [33,43]
13 ^a^	‘IV9 Calcutta4’ (AA)	MRF526	-	R [33]	R [33]
14 ^a^	‘Pisang Jari Buaya’ (AA)	MRF1244	-	R [33]	R [33,45]
15 ^a^	‘Ma-ITC0250’ (malaccensis)	MRF826	+(Het)	R [33]	R [33]
16 ^a^	‘M61 Guadeloupe’ (AA)	MRF654	-	SS [33]	R [33]
17 ^a^	‘CAM-020’ (AA)	MRF1657	-	S [33]	R [33]
18 ^a^	‘SH-3142’ (AA)	MRF1984	-	R [33]	R [33,43]
19 ^a^	*M. a. malaccensis*	ITC0399	+(Het)	n/a	n/a
20 ^a^	‘Pahang’ (malaccensis)	ITC0609	+	R [33]	R [33,40,45,60]
21 ^b^	‘Pa Musore no2’ (*M. acuminata* spp.)	ITC0668	+(Het)	n/a	n/a
22 ^b^	‘Kluai Pal’ (malaccensis)	ITC0979	+(Het)	n/a	n/a
23 ^b^	‘DH Pahang’ (malaccensis)	ITC1511	+	n/a	R [45,46]
24 ^b^	*M a. malaccensis*	ITC0074	-	n/a	n/a
25 ^b^	‘Pa Musore no3’ (*M. acuminata* spp.)	ITC0406	-	n/a	n/a
26 ^b^	‘Pa_Songkhla’ (*M. acuminata* spp.)	ITC0408	-	n/a	n/a
27 ^b^	‘Selangor 2’ (malaccensis)	ITC0629	-	n/a	n/a
28 ^b^	‘Pisang Raja Udang’ (AA)	ITC0976	-	n/a	n/a
29 ^b^	‘THA018’ (malaccensis)	ITC1067	-	n/a	n/a
30 ^b^	‘Pisang Kra’ (malaccensis)	ITC1345	-	n/a	n/a
31 ^b^	‘Pisang Serun 403’ (malaccensis)	ITC1347	-	n/a	n/a
32 ^b^	‘Pisang Serun 404’ (malaccensis)	ITC1348	-	n/a	n/a
33 ^b^	‘Pisang Serun 400’ (malaccensis)	ITC1349	-	n/a	n/a
34 ^b^	‘IB-99’	ITC1447	-	n/a	n/a
35 ^c^	‘TMB2×7197-2’ (AA)	-	+(Het)	n/a	n/a
36 ^c^	‘5610S-1’ (AA)	-	+(Het)	n/a	n/a
37 ^c^	‘SH-3362’ (AA)	MUSA214	+(Het)	R [33]	R [33]
38 ^c^	‘Malaccensis 250’ (malaccensis)	ITC0250	+(Het)	R [33]	n/a
39 ^c^	‘SH-3217’ (AA)	MMC218	+	R [33]	R [43]
40 ^c^	‘SH-3361’ (AA)	-	+(Het)	n/a	n/a
41 ^c^	‘TMB2×8075-7’ (AA)	-	-	n/a	n/a
42 ^c^	‘Hutishamba’ (AA)	MMC486	-	n/a	n/a
43 ^c^	‘Mshare Laini’ (AA)	-	-	n/a	n/a
44 ^c^	‘cv. Rose’ (AA)	ITC0712	-	n/a	R [40,41]
45 ^c^	‘Mularu’ (AA)	MMC465	-	n/a	n/a
46 ^c^	‘Kamunyila’ (AA)	MMC479	-	n/a	n/a
47 ^c^	‘Mlelembo’ (AA)	ITC1544	-	n/a	n/a
48 ^c^	‘Njuru’ (AA)	MMC418	-	n/a	n/a
49 ^c^	‘Kahuti’ (AA)	ITC1468	-	n/a	n/a
50 ^c^	‘Mbwazirume’ (AAA)	ITC0084	-	n/a	R [45]
51 ^c^	‘Sukari Ndiizi’ (AAB)	MMC167	-	n/a	n/a
52 ^c^	‘Nshonowa’ (AA)	ITC1466	-	n/a	n/a
53 ^d^	‘FHIA-3’ (AABB)	MRF1941	+(Het)	S [33,61]	SS [33], S [41], R [43]
54 ^d^	‘FHIA-25’ (AAB)	MRF1960	+	R [33]	R [33,43,45]
55 ^d^	‘FHIA-21’ (AAAB)	MRF1205	-	n/a	S [41], R [45]
56 ^d^	‘FHIA-23’ (AAAA)	MRF1207	-	S [33]	SS [33], S [41]
57 ^d^	‘GCTCV-119’ (AAA)	MRF1860	-	R [33]	R [33,41]
58 ^d^	‘FHIA-2’ (AAAB)	MRF1933	-	S [33,61]	R [33,43], S [41]
59 ^d^	‘FHIA-1’/’Goldfinger’ (AAAB)	MRF1959	-	R [33]	R [33,43], S [62]
60 ^d^	*Musa balbisiana* (BB)	MRF1593	-	S [33]	S [62]

## Data Availability

All data analysed during this study are included in this article and its supplementary files. The RNAseq data described in this study are available on request from the corresponding author. They are not publicly available due to confidentiality of genetic information pertaining to the gene discovery.

## Supplementary Materials

The following supporting information can be downloaded at: https://www.mdpi.com/article/10.3390/pathogens12060820/s1, Table S1: ‘DH Pahang’ v4 gene models within the candidate region; Table S2: Enrichment of Gene Ontology (GO) terms detected in the candidate region using p and q cutoffs of 0.05 and 0.1, respectively; Table S3: Screening of the IITA germplasm collection (Uganda) using the A-genome-specific marker 29730-A; Table S4: IITA *Musa acuminata* ssp. *banksii* collection from Ibadan, Nigeria, screened with the CAPS marker, 29730.
