# Supplementary material for: Genetic Mapping, Candidate Gene Identification and Marker Validation for Host Plant Resistance to the Race 4 of Fusarium oxysporum f. sp. cubense Using Musa acuminata ssp. malaccensis
Source: Pathogens. 2023 Jun 9;12(6):820. doi: 10.3390/pathogens12060820 (PMC10303076; doi:10.3390/pathogens12060820)
Supplement: Supplementary file 1 [file pathogens-12-00820-s001.zip › pathogens-2405012-supplementary.pdf]

Genetic Mapping, Candidate Gene Identification and Marker Validation for Host Plant Resistance to the Race 4 of *Fusarium oxysporum* f. sp. *cubense* using *Musa acuminata* ssp. *malaccensis*

Supplementary Data

Table S1

Table S2

Table S3.

Table S4.

Table S1. 'DH Pahang' v4 gene models within the candidate region. Bold highlighted gene models were used as CAPS markers for mapping. An asterisk (*) indicates the differentially expressed genes presented in **Figure 4**.

'DH-Pahang' v4	Chr3 position	Description
**Macma4_03_g31200.1 (Marker 28420)**	**41185133..41196344**	**F-box domain-containing protein**
Macma4_03_g31210.1	41206680..41207872	Phytocyanin domain-containing protein
Macma4_03_g31220.1	41211260..41251694	DNA-directed RNA polymerase I subunit 1
Macma4_03_g31230.1	41253561..41255269	SAE2 domain-containing protein
Macma4_03_g31240.1	41260568..41266884	60S ribosomal protein L6
Macma4_03_g31250.1	41267214..41271887	Protein kinase domain-containing protein
Macma4_03_g31260.1	41272437..41277100	Phosphoenolpyruvate/phosphate translocator 1, chloroplastic
Macma4_03_g31270.1	41277244..41277947	PMEI domain-containing protein
Macma4_03_g31280.1	41279040..41281384	Conserved hypothetical protein
Macma4_03_g31290.1	41282123..41290354	Farnesyl pyrophosphare synthase
Macma4_03_g31300.1	41295141..41296569	Myb-related protein Hv33
Macma4_03_g31310.1*	41300083..41303456	LRRNT_2 domain-containing protein
Macma4_03_g31320.1*	41385591..41449859	LRR receptor-like serine/threonine-protein kinase GSO1
Macma4_03_g31330.1	41320808..41323526	Cysteine-rich receptor-like protein kinase 6
Macma4_03_g31340.1	41312026..41315013	LRRNT_2 domain-containing protein
Macma4_03_g31350.1	41333081..41346645	LRR receptor-like serine/threonine-protein kinase GSO1
Macma4_03_g31360.1	41356337..41359555	LRRNT_2 domain-containing protein
Macma4_03_g31370.1	41351929..41352780	Receptor-like protein EIX2
Macma4_03_g31380.1*	41364558..41367455	LRRNT_2 domain-containing protein
Macma4_03_g31390.1	41369977..41396220	Transcription factor MYB86
Macma4_03_g31400.1	41373362..41377132	LRRNT_2 domain-containing protein
Macma4_03_g31410.1	41414485..41425094	Receptor-like protein EIX2
Macma4_03_g31420.1	41395720..41413012	Conserved hypothetical protein

Macma4_03_g31430.1	41408283..41409167	Cysteine-rich repeat secretory protein 38
Macma4_03_g31440.1	41428541..41430963	Hypothetical protein
Macma4_03_g31450.1	41431234..41435561	Receptor-like protein EIX2
Macma4_03_g31460.1*	41459414..41497257	Receptor-like protein EIX2
Macma4_03_g31470.1*	41517334..41520795	Receptor-like protein EIX2
Macma4_03_g31480.1	41472994..41475737	Cysteine-rich receptor-like protein kinase 6
Macma4_03_g31490.1	41489067..41490234	Myb-related protein Hv33
Macma4_03_g31500.1	41508341..41511598	Receptor-like protein EIX2
Macma4_03_g31510.1*	41524565..41527279	Cysteine-rich receptor-like protein kinase 6
Macma4_03_g31520.1	41544749..41548495	Receptor-like protein EIX2
Macma4_03_g31530.1	41532334..41535839	Receptor-like protein EIX2
Macma4_03_g31540.1	41566336..41569054	Cysteine-rich receptor-like protein kinase 6
Macma4_03_g31550.1	41555821..41593102	Receptor-like protein EIX2
Macma4_03_g31560.1	41595512..41595742	Hypothetical protein
Macma4_03_g31570.1	41602983..41606582	Receptor-like protein EIX2
Macma4_03_g31580.1	41622231..41625464	Receptor-like protein EIX2
Macma4_03_g31590.1	41629507..41639565	Receptor-like protein EIX2
Macma4_03_g31600.1	41641838..41656824	Hypothetical protein
Macma4_03_g31610.1	41645278..41648736	Receptor-like protein EIX2
Macma4_03_g31620.1	41652520..41655238	Cysteine-rich receptor-like protein kinase 6
Macma4_03_g31630.1	41658517..41661609	Receptor-like protein EIX2
Macma4_03_g31640.1	41671331..41674950	Receptor-like protein EIX2
Macma4_03_g31650.1	41677256..41678423	Myb-related protein Hv33
Macma4_03_g31660.1	41683972..41687720	Receptor-like protein EIX2
Macma4_03_g31670.1	41690735..41691128	Hypothetical protein
**Macma4_03_g31680.1 (Marker 28820)**	**41695490..41699989**	**Bifunctional nuclease 2**
Macma4_03_g31690.1	41701523..41712616	Phosphatidylinositol/phosphatidylcholine transfer protein SFH8
Macma4_03_g31700.1	41715069..41719865	Hypothetical protein
Macma4_03_g31710.1	41723662..41727708	Mitogen-activated protein kinase kinase kinase 18
Macma4_03_g31720.1	41726550..41729437	Hypothetical protein
Macma4_03_g31730.1	41730302..41734414	Zinc finger BED domain-containing protein RICESLEEPER 1
Macma4_03_g31740.1	41734749..41737252	Pentatricopeptide repeat-containing protein At4g04370
Macma4_03_g31750.1	41738751..41742387	Hypothetical protein
Macma4_03_g31760.1	41742883..41744394	Hypothetical protein
Macma4_03_g31770.1	41746163..41747718	Two-pore potassium channel 5
Macma4_03_g31780.1	41751185..41752315	Hypothetical protein
Macma4_03_g31790.1	41752658..41762344	Glycosyltransferase BC10
Macma4_03_g31800.1	41766079..41766204	Hypothetical protein
Macma4_03_g31810.1	41772196..41775831	Ubiquitin-conjugating enzyme E2 5A
Macma4_03_g31820.1	41776216..41779883	Protein GAMETE EXPRESSED 3
Macma4_03_g31830.1	41782445..41798498	Conserved hypothetical protein
Macma4_03_g31840.1	41799341..41799785	Hypothetical protein
Macma4_03_g31850.1	41800716..41803719	Serine/threonine-protein kinase/endoribonuclease IRE1a
Macma4_03_g31860.1	41806710..41809547	Serine/threonine-protein kinase/endoribonuclease IRE1
Macma4_03_g31870.1	41805410..41806709	Hypothetical protein
Macma4_03_g31880.1	41810853..41811823	Conserved hypothetical protein

Macma4_03_g31890.1	41812631..41813493	Conserved hypothetical protein
Macma4_03_g31900.1	41814473..41815783	Hypothetical protein
Macma4_03_g31910.1	41815696..41816622	Hypothetical protein
Macma4_03_g31920.1	41822154..41823333	Hypothetical protein
Macma4_03_g31930.1	41824383..41832871	Notchless protein
Macma4_03_g31940.1	41832872..41837859	Conserved hypothetical protein
Macma4_03_g31950.1	41833882..41835411	Palmitoyl-acyl carrier protein thioesterase, chloroplastic
Macma4_03_g31960.1	41839873..41845205	Protein DETOXIFICATION 10
Macma4_03_g31970.1	41847431..41852161	Hypothetical protein
Macma4_03_g31980.1	41852762..41857256	ATP synthase subunit beta, mitochondrial
Macma4_03_g31990.1	41861903..41862442	Hypothetical protein
Macma4_03_g32000.1	41864457..41867377	Ribose-5-phosphate isomerase 2
Macma4_03_g32010.1	41871539..41879072	Hypothetical protein
Macma4_03_g32020.1	41880517..41886745	AUGMIN subunit 2
Macma4_03_g32030.1	41890891..41893288	IQ domain-containing protein IQM2
Macma4_03_g32040.1	41893861..41895567	Pentatricopeptide repeat-containing protein At4g39530
Macma4_03_g32050.1*	41900067..41909547	Conserved hypothetical protein
Macma4_03_g32060.1	41897661..41900656	Conserved hypothetical protein
Macma4_03_g32070.1	41909935..41916078	Conserved hypothetical protein
Macma4_03_g32080.1	41916897..41925044	Conserved hypothetical protein
Macma4_03_g32090.1	41930452..41935037	Conserved hypothetical protein
Macma4_03_g32100.1	41935101..41945359	Protein NAR1
Macma4_03_g32110.1	41947092..41948011	Ethylene-responsive transcription factor 9
Macma4_03_g32120.1	41949180..41971744	Protein WRKY1
Macma4_03_g32130.1	41972765..41975625	Transcription factor bHLH92
Macma4_03_g32140.1	41990262..41997002	CRM-domain containing factor CFM3, chloroplastic/mitochondrial
Macma4_03_g32150.1	41997575..41998435	PRA1 family protein B5
Macma4_03_g32160.1	41998701..42010823	Glutathione reductase, chloroplastic
Macma4_03_g32170.1	42012734..42015779	Hypothetical protein
Macma4_03_g32180.1	42017982..42019326	AP2/ERF and B3 domain-containing transcription repressor RAV2
Macma4_03_g32190.1	42023042..42026327	Conserved hypothetical protein
Macma4_03_g32200.1	42028072..42031234	Receptor protein kinase-like protein ZAR1
Macma4_03_g32210.1	42033960..42035510	Glutamine amidotransferase GAT1_2.1
Macma4_03_g32220.1*	42035600..42038030	*LEAF RUST 10 DISEASE-RESISTANCE LOCUS RECEPTOR-LIKE PROTEIN KINASE-like 2.1*
Macma4_03_g32230.1	42038571..42039543	*LEAF RUST 10 DISEASE-RESISTANCE LOCUS RECEPTOR-LIKE PROTEIN KINASE-like 1.2*
Macma4_03_g32240.1	42043048..42045672	*LEAF RUST 10 DISEASE-RESISTANCE LOCUS RECEPTOR-LIKE PROTEIN KINASE-like 2.1*
Macma4_03_g32250.1	42046320..42047515	Hypothetical protein
Macma4_03_g32260.1	42048539..42049555	*LEAF RUST 10 DISEASE-RESISTANCE LOCUS RECEPTOR-LIKE PROTEIN KINASE-like 1.2*
Macma4_03_g32270.1	42052018..42058909	*LEAF RUST 10 DISEASE-RESISTANCE LOCUS RECEPTOR-LIKE PROTEIN KINASE-like 1.3*
Macma4_03_g32280.1	42052949..42054869	*LEAF RUST 10 DISEASE-RESISTANCE LOCUS RECEPTOR-LIKE PROTEIN KINASE-like 1.2*
Macma4_03_g32290.1	42059396..42062500	Universal stress protein A-like protein
Macma4_03_g32300.1	42063437..42064515	Hypothetical protein
Macma4_03_g32310.1	42065532..42066512	Adenylate isopentenyltransferase

Macma4_03_g32320.1	42070221..42078056	Caffeoylshikimate esterase
Macma4_03_g32330.1	42078115..42085871	Hypothetical protein
Macma4_03_g32340.1	42088311..42089937	LOB domain-containing protein 41
Macma4_03_g32350.1	42089938..42092882	Hypothetical protein
Macma4_03_g32360.1	42098922..42099597	Ethylene-responsive transcription factor ERF070
Macma4_03_g32370.1	42099885..42102752	Beta-glucosidase 1
Macma4_03_g32380.1	42105068..42106673	Conserved hypothetical protein
Macma4_03_g32390.1	42110299..42112091	Hypothetical protein
Macma4_03_g32400.1	42118206..42119901	Receptor-like protein EIX2
Macma4_03_g32410.1	42121987..42124957	F-box/kelch-repeat protein At1g55270
Macma4_03_g32420.1	42125460..42130238	Peptidyl-tRNA hydrolase, mitochondrial
Macma4_03_g32430.1	42131732..42137853	Eukaryotic translation initiation factor NCBP
**Macma4_03_g32440.1 (Marker 29590)**	**42138268..42142592**	**Pentatricopeptide repeat-containing protein At4g28010**

Table S2. Enrichment of Gene Ontology (GO) terms detected in the candidate region using p and q cutoffs of 0.05 and 0.1, respectively. Using the GO tool at 'https://banana-genome-hub.southgreen.fr/content/go-enrichment' (accessed on 21 April 2023), 102 out of the 125 candidate gene models in DH-Pahang v4 (26,613 genes and 122,637 GO terms) are associated with at least 1 GO term. The gene models belonging to each GO term are listed, along with the gene ratio and the corresponding p-adj value. An asterisk (*) indicates the differentially expressed genes presented in **Figure 4**.

**Biological processes**	**Description**	**Gene Ratio**	**p-adj**
**GO:0042742**	**Defense response to bacterium**	**7/102**	**0.00043759**
Macma4_03_g31330.1	Cysteine-rich receptor-like protein kinase 6		
Macma4_03_g31430.1	Cysteine-rich repeat secretory protein 38		
Macma4_03_g31480.1	Cysteine-rich receptor-like protein kinase 6		
Macma4_03_g31510.1*	Cysteine-rich receptor-like protein kinase 6		
Macma4_03_g31540.1	Cysteine-rich receptor-like protein kinase 6		
Macma4_03_g31620.1	Cysteine-rich receptor-like protein kinase 6		
Macma4_03_g31850.1	Serine/threonine-protein kinase/endoribonuclease IRE1a		
**GO:0030968**	**Endoplasmic reticulum unfolded protein response**	**3/102**	**0.00192488**
Macma4_03_g31850.1	Serine/threonine-protein kinase/endoribonuclease IRE1a		
Macma4_03_g31860.1	Serine/threonine-protein kinase/endoribonuclease IRE1		
Macma4_03_g32050.1*	Conserved hypothetical protein		
**GO:0050832**	**Defense response to fungus**	**5/102**	**0.03443587**
Macma4_03_g31370.1	Receptor-like protein EIX2		
Macma4_03_g31410.1	Receptor-like protein EIX2		
Macma4_03_g31450.1	Receptor-like protein EIX2		
Macma4_03_g31590.1	Receptor-like protein EIX2		
Macma4_03_g32400.1	Receptor-like protein EIX2		
**GO:0071215**	**Cellular response to abscisic acid stimulus**	**2/102**	**0.04062588**
Macma4_03_g32230.1	*LEAF RUST 10 DISEASE-RESISTANCE LOCUS RECEPTOR-LIKE PROTEIN KINASE-like 1.2*		
Macma4_03_g32280.1	*LEAF RUST 10 DISEASE-RESISTANCE LOCUS RECEPTOR-LIKE PROTEIN KINASE-like 1.2*		

Molecular function	Description	Gene Ratio	p-adj
**GO:0030247**	**Polysaccharide binding**	**7/102**	**9.43E-06**
Macma4_03_g32220.1*	*LEAF RUST 10 DISEASE-RESISTANCE LOCUS RECEPTOR-LIKE PROTEIN KINASE-like 2.1*		
Macma4_03_g32230.1	*LEAF RUST 10 DISEASE-RESISTANCE LOCUS RECEPTOR-LIKE PROTEIN KINASE-like 1.2*		
Macma4_03_g32240.1	*LEAF RUST 10 DISEASE-RESISTANCE LOCUS RECEPTOR-LIKE PROTEIN KINASE-like 2.1*		
Macma4_03_g32250.1	Hypothetical protein		
Macma4_03_g32260.1	*LEAF RUST 10 DISEASE-RESISTANCE LOCUS RECEPTOR-LIKE PROTEIN KINASE-like 1.2*		
Macma4_03_g32270.1	*LEAF RUST 10 DISEASE-RESISTANCE LOCUS RECEPTOR-LIKE PROTEIN KINASE-like 1.3*		
Macma4_03_g32280.1	*LEAF RUST 10 DISEASE-RESISTANCE LOCUS RECEPTOR-LIKE PROTEIN KINASE-like 1.2*		
**GO:0004521**	**Endoribonuclease activity**	**3/102**	**0.00586628**
Macma4_03_g31850.1	Serine/threonine-protein kinase/endoribonuclease IRE1a		
Macma4_03_g31860.1	Serine/threonine-protein kinase/endoribonuclease IRE1		
Macma4_03_g32050.1*	Conserved hypothetical protein		

Table S3. Screening of the IITA germplasm collection (Uganda) using the A-genome specific marker 29730-A (**Table 2**). Genome subgroup and ploidy is indicated in brackets. F- and M-parent respectively indicate female and male parent used in the crossing. NARITA hybrids are East African Highland Bananas (EAHBs) named after the organizations involved in the collaborative development of these lines, namely the National Agricultural Research Organization (NARO) of Uganda and the International Institute of Tropical Agriculture (IITA). SH stands for selected hybrids developed by breeding program at the Honduran Agricultural Research Foundation (Fundación Hondureña de Investigación Agrícola or FHIA), Honduras. 29730-A marker alleles were scored as per previous description (Table 2). A = marker allele for homozygous resistance, B = marker allele for homozygous susceptibility, H = heterozygous.

sn	Genotype	Type	F-Parent	M-Parent	A-29730
1	1201K-1 (AAAA)	Matooke hybrid (Parental)	Nakawere (AAA)	Calcutta 4 (AA)	B
2	1438K-1 (AAAA)	Matooke hybrid (Parental)	Entukura (AAA)	Calcutta 4 (AA)	H
3	222K-1 (AAAA)	Matooke hybrid (Parental)	Nfuuka (AAA)	Calcutta 4 (AA)	B
4	365K-1(AAAA)	Matooke hybrid (Parental)	Kabucuragye (AAA)	Calcutta 4 (AA)	B
5	376K-7 (AAAA)	Matooke hybrid (Parental)	Nante (AAA)	Calcutta 4 (AA)	H
6	401K-1 (AAAA)	Matooke hybrid (Parental)	Entukura (AAA)	Calcutta 4 (AA)	B
7	660K-1 (AAAA)	Matooke hybrid (Parental)	Enzirabahima (AAA)	Calcutta 4 (AA)	B
8	NARITA 1 (AAA)	Maooke hybrid (NARITA)	917K-2 (AAAA)	TMB2×9128-3 (AA)	H
9	NARITA 2 (AAA)	Maooke hybrid (NARITA)	401K-1 (AAAA)	TMB2×9128-3 (AA)	B
10	NARITA 3 (AAA)	Maooke hybrid (NARITA)	917K-2 (AAAA)	SH3362 (AA)	B
11	NARITA 4 (AAA)	Maooke hybrid (NARITA)	660K-1 (AAAA)	TMB2×9128-3 (AA)	B
12	NARITA 5 (AAA)	Maooke hybrid (NARITA)	917K-2 (AAAA)	SH3217 (AA)	H
13	NARITA 6 (AAA)	Maooke hybrid (NARITA)	222K-1 (AAAA)	TMB2×9128-3 (AA)	H
14	NARITA 7 (AAA)	Maooke hybrid (NARITA)	1201K-1 (AAAA)	SH3217 (AA)	H

15	NARITA 8 (AAA)	Maooke hybrid (NARITA)	917K-2 (AAAA)	SH3217 (AA)	H
16	NARITA 9 (AAA)	Maooke hybrid (NARITA)	917K-2 (AAAA)	SH3217 (AA)	H
17	NARITA 10 (AAA)	Maooke hybrid (NARITA)	917K-2 (AAAA)	SH3217 (AA)	-
18	NARITA 11 (AAA)	Maooke hybrid (NARITA)	1201K-1 (AAAA)	TMB2×9128-3 (AA)	B
19	NARITA 12 (AAA)	Maooke hybrid (NARITA)	1201K-1 (AAAA)	TMB2×9128-3 (AA)	B
20	NARITA 13 (AAA)	Maooke hybrid (NARITA)	1201K-1 (AAAA)	SH3362 (AA)	H
21	NARITA 14 (AAA)	Maooke hybrid (NARITA)	917K-2 (AAAA)	TMB2×7197-2 (AA)	B
22	NARITA 15 (AAA)	Maooke hybrid (NARITA)	660K-1 (AAAA)	TMB2×9128-3 (AA)	H
23	NARITA 16 (AAA)	Maooke hybrid (NARITA)	917K-2 (AAAA)	SH3362 (AA)	H
24	NARITA 17 (AAA)	Maooke hybrid (NARITA)	1438K-1 (AAAA)	TMB2×9719-7 (AA)	H
25	NARITA 18 (AAA)	Maooke hybrid (NARITA)	365K-1 (AAAA)	660K-1 (AAAA)	B
26	NARITA 19 (AAA)	Maooke hybrid (NARITA)	1201K-1 (AAAA)	TMB2×8075-7 (AA)	H
27	NARITA 20 (AAA)	Maooke hybrid (NARITA)	Entukura (AAA)	365K-1 (AAAA)	B
28	NARITA 21 (AAA)	Maooke hybrid (NARITA)	1201K-1 (AAAA)	TMB2×7197-2 (AA)	B
29	NARITA 22 (AAA)	Maooke hybrid (NARITA)	917K-2 (AAAA)	TMB2×9128-3 (AA)	H
30	NARITA 23 (AAA)	Maooke hybrid (NARITA)	Kazirakwe (AAA)	TMB2×7197-2 (AA)	H
31	NARITA 24 (AAA)	Maooke hybrid (NARITA)	Unknown	Unknown	B
32	NARITA 25 (AAA)	Maooke hybrid (NARITA)	Unknown	Unknown	H
33	NARITA 26 (AAA)	Maooke hybrid (NARITA)	Unknown	Unknown	B
34	24948S-10 (AAA)	Matooke hybrid	1438K-1 (AAAA)	5610S-1 (AA)	H
35	24948S-9 (AAA)	Matooke hybrid	1438K-1 (AAAA)	5610S-1 (AA)	H
36	25356S-1 (AAA)	Matooke hybrid	Tereza (AAA)	TMB2×7197-2 (AA)	H
37	25583S-2 (AAA)	Matooke hybrid	1201K-1 (AAAA)	5610S-1 (AA)	H
38	25623S-11 (AAA)	Matooke hybrid	8817S-1 (AA)	917K-2 (AAAA)	H
39	25909S-3 (AAA)	Matooke hybrid	917K-2 (AAAA)	TMB2×7197-2 (AA)	H
40	25974S-30 (AAA)	Matooke hybrid	917K-2 (AAAA)	SH3362 (AA)	H
41	25974S-31 (AAA)	Matooke hybrid	917K-2 (AAAA)	SH3362 (AA)	H
42	26260S-3 (AAA)	Matooke hybrid	660K-1 (AAAA)	5610S-1 (AA)	H
43	26316S-7 (AAA)	Matooke hybrid	1201K-1 (AAAA)	SH3362 (AA)	H
44	26337S-37 (AAA)	Matooke hybrid	1201K-1 (AAAA)	SH3217 (AA)	H
45	26666S-1 (AAA)	Matooke hybrid	917K-2 (AAAA)	SH3362 (AA)	B
46	26815S-3 (AAA)	Matooke hybrid	917K-2 (AAAA)	5610S-1 (AA)	B
47	27401S-1 (AAA)	Matooke hybrid	917K-2 (AAAA)	Malaccensis_250 (AA)	B
48	27579S-1 (AAA)	Matooke hybrid	1201K-1 (AAAA)	Malaccensis_250 (AA)	B
49	27825S-4 (AAA)	Matooke hybrid	660K-1 (AAAA)	Malaccensis_250 (AA)	B
50	27873S-26 (AAA)	Matooke hybrid	660K-1 (AAAA)	Malaccensis_250 (AA)	B
51	27873S-38 (AAA)	Matooke hybrid	660K-1 (AAAA)	Malaccensis_250 (AA)	B
52	28033S-19 (AAA)	Matooke hybrid	917K-2 (AAAA)	Malaccensis_250 (AA)	H
53	28033S-23 (AAA)	Matooke hybrid	917K-2 (AAAA)	Malaccensis_250 (AA)	B
54	28060S-5 (AAA)	Matooke hybrid	917K-2 (AAAA)	Malaccensis_250 (AA)	B
55	28060S-8 (AAA)	Matooke hybrid	917K-2 (AAAA)	Malaccensis_250 (AA)	B
56	28257S-1 (AAA)	Matooke hybrid	917K-2 (AAAA)	Malaccensis_250 (AA)	H
57	28434S-2 (AAA)	Matooke hybrid	1201K-1 (AAAA)	5610S-1 (AA)	B
58	28465S-21 (AAA)	Matooke hybrid	1201K-1 (AAAA)	Malaccensis_250 (AA)	H
59	28974S-11 (AAA)	Matooke hybrid	1438K-1 (AAAA)	Malaccensis_250 (AA)	H
60	29114S-14 (AAA)	Matooke hybrid	5610S-1 (AA)	Malaccensis_250 (AA)	H
61	29114S-24 (AAA)	Matooke hybrid	5610S-1 (AA)	Malaccensis_250 (AA)	H
62	29275S-1 (AAAA)	Matooke hybrid (Parental)	Enzirabahima (AAA)	Malaccensis_250 (AA)	B
63	29275S-5 (AAAA)	Matooke hybrid (Parental)	Enzirabahima (AAA)	Malaccensis_250 (AA)	B

64 29364S-2 (AAAA)	Matooke hybrid (Parental)	Namwezi (AAA)	Cv. Rose (AA)	B
65 29490S-1 (AAA)	Matooke hybrid	917K-2 (AAAA)	5610S-1 (AA)	H
66 29561S-2 (AAA)	Matooke hybrid	1201K-1 (AAAA)	Malaccensis_250 (AA)	B
67 29586S-4 (AAA)	Matooke hybrid	1438K-1 (AAAA)	5610S-1 (AA)	H
68 29275S-4 (AAAA)	Matooke hybrid (Parental)	Enzirabahima (AAA)	Malaccensis_250 (AA)	B
69 Zebrina (G.F.) (AA)	Diploid (Parental)	*Musa acuminata* ssp. *zebrina*		B
70 TMB2×5265-1 (AA)	Diploid (Parental)	Tjau lagada (AA)	Calcutta 4 (AA)	B
71 02145/1320 (AA)	Diploid (Parental)	Zebrina GF	Open pollination	B
72 10969S-1 (AA)	Diploid (Parental)	376K-7 (AAAA)	5105-1 (AA)	B

Table S4. IITA *Musa acuminata* ssp. *banksii* collection from Ibadan, Nigeria, screened with the CAPS marker 29730. Marker alleles are described as per **Table S1**. [a]phylogenetic grouping refers to the unweighted pair group method with arithmetic mean (UPGMA) clustering of these accessions in a previously published phylogenetic analysis on *Musa* diversity [59].

sn	Accession name	ITC code	Phylogenetic grouping[a]	A-29730
1	Galeo	ITC0259	AA cv. IndonTriNG	B
2	Sowmuk	ITC0266	AA cv. banksii sensu lato	B
3	NBA 14	ITC0267	AA cv. banksii sensu lato	B
4	Niyarma Yik	ITC0269	AA cv. banksii sensu lato	B
5	Pitu	ITC0294	AA cv. IndonTriNG	B
6	Beram	ITC0298	AA cv. IndonTriNG	B
7	Guyod	ITC0299	AA cv. IndonTriPh	B
8	UPLB	ITC0343	AA cv. banksii sensu lato	B
9	Higa	ITC0428	*M. acuminata* ssp. *banksii*	B
10	Banksii	ITC0453	*M. acuminata* ssp. *banksii*	B
11	Waimara	ITC0600	AA cv. banksii derivative	B
12	Hung Tu	ITC0601	AA cv. IndonTriNG	B
13	Hawain 3	ITC0602	*M. acuminata* ssp. *banksii*	B
14	Somani	ITC0603	AA cv. banksii sensu lato	B
15	Hybrid	ITC0606	*M. acuminata* ssp. *banksii*	B
16	Mambee Thu	ITC0612	AA cv. IndonTriNG	B
17	Hawain 3	ITC0617	*M. acuminata* ssp. *banksii*	B
18	Banksii	ITC0620	*M. acuminata* ssp. *banksii*	B
19	Navaradam	ITC0770	AA cv. banksii derivative	B
20	Mpiajhap	ITC0773	AA cv. banksii derivative	B
21	Garunga	ITC0798	AA cv. banksii derivative	B
22	Banksii	ITC0806	*M. acuminata* ssp. *banksii*	B
23	Maleb	ITC0809	AA cv. banksii sensu lato	B
24	Sihir	ITC0810	AA cv. banksii derivative	B
25	Sepi	ITC0849	AA cv. banksii sensu lato	B
26	Mala	ITC0869	AA cv. IndonTriNG	B
27	Banksii	ITC0879	*M. acuminata* ssp. *banksii*	B
28	Kwosriake	ITC0882	AA cv. banksii sensu lato	B
29	Awondaeke	ITC0884	AA cv. IndonTriNG	B

30	Banksii	ITC0885	*M. acuminata* ssp. *banksii*	B
31	Wikago	ITC0888	AA cv. banksii sensu lato	B
32	Pai ka	ITC0892	AA cv. banksii sensu lato	B
33	Adina	ITC0893	AA cv. banksii sensu lato	B
34	Tainga	ITC0894	AA cv. banksii derivative	B
35	Fu Des	ITC0939	AA cv. IndonTriNG	B
36	Kwaro	ITC0943	AA cv. IndonTriNG	B
37	Tomolo	ITC1187	AA cv. banksii sensu lato	B
38	Spiral	ITC1206	AA cv. banksii derivative	B
39	Banksii	ITC1219	*M. acuminata* ssp. *banksii*	B
40	Kokopo	ITC1243	AA cv. IndonTriNG	B
41	Mapua	ITC1244	AA cv. banksii sensu lato	B
42	SH3362	n.a.	2x hybrid parent	H
43	TMB2×8075-7	n.a.	2x hybrid parent	B
44	30456-1	n.a.	3x BITA hybrid	B
45	30456-2	n.a.	3x BITA hybrid	B
46	30456-3	n.a.	4x BITA hybrid	B

59. Christelová, P.; De Langhe, E.; Hřibová, E.; Čížková, J.; Sardos, J.; Hušáková, M.; Van den houwe, I.; Sutanto, A.; Kepler, A.K.; Swennen, R.; et al. Molecular and cytological characterization of the global *Musa* germplasm collection provides insights into the treasure of banana diversity. *Biodivers. Conserv.* **2017**, *26*, 801–824. https://doi.org/10.1007/s10531-016-1273-9.
